# Identification of the SNARE complex that mediates the fusion of multivesicular bodies with the plasma membrane in exosome secretion

**DOI:** 10.1002/jev2.12356

**Published:** 2023-09-12

**Authors:** Chuqi Liu, Dexiang Liu, Shen Wang, Lu Gan, Xiangliang Yang, Cong Ma

**Affiliations:** ^1^ Key Laboratory of Molecular Biophysics of the Ministry of Education, College of Life Science and Technology Huazhong University of Science and Technology Wuhan China; ^2^ National Engineering Research Center for Nanomedicine, College of Life Science and Technology Huazhong University of Science and Technology Wuhan China; ^3^ Hubei Key Laboratory of Bioinorganic Chemistry and Materia Medica Huazhong University of Science and Technology Wuhan China; ^4^ Hubei Key Laboratory of Bioinorganic Chemistry and Materia Medica Huazhong University of Science and Technology Wuhan China; ^5^ GBA Research Innovation Institute for Nanotechnology Guangzhou Guangdong P. R. China

**Keywords:** breast cancer, exosome secretion, membrane fusion, multivesicular bodies, SNARE

## Abstract

Exosomes play crucial roles in local and distant cellular communication and are involved in various physiological and pathological processes. Tumour‐derived exosomes are pivotal to tumorigenesis, but the precise mechanisms underlying their secretion remain elusive. In particular, the SNARE proteins that mediate the fusion of multivesicular bodies (MVBs) with the plasma membrane (PM) in tumour cells are subject to debate. In this study, we identified syntaxin‐4, SNAP‐23, and VAMP‐7 as the SNAREs responsible for exosome secretion in MCF‐7 breast cancer cells and found that a SNARE complex consisting of these SNAREs can drive membrane fusion in vitro. Deletion of any of these SNAREs in MCF‐7 cells did not affect MVB biogenesis and transportation, indicating their specific involvement in MVB–PM fusion. In addition, syntaxin‐4, SNAP‐23, and VAMP‐7 play equivalent roles in exosome secretion in both HeLa cervical cancer cells and A375 melanoma cells, suggesting their conserved function in exosome secretion. Furthermore, deletion of VAMP‐7 in 4T1 mammary carcinoma cells efficiently inhibited exosome secretion and led to significant attenuation of tumour growth and lung metastasis in mouse models, implying that VAMP‐7 may hold promise as a novel therapeutic target for breast cancer.

## 1 INTRODUCTION

Exosomes, which range from 30 to 150 nm in diameter, are single‐membrane extracellular vesicles secreted by almost all types of cells. These vesicles contain a selective assortment of proteins, lipids, nucleic acids, glycoconjugates, and metabolites (Gurung et al., 2021; Kalluri & LeBleu, 2020). Exosomes originate as intraluminal vesicles (ILVs) that form through the inward budding of the endosomal membrane during the maturation of multivesicular endosomes (MVEs; also called multivesicular bodies [MVBs]). Upon fusion of MVBs with the plasma membrane (PM), the ILVs are secreted into the extracellular space (Colombo et al., 2014; Huotari & Helenius, 2011; van Niel et al., 2018). Once secreted, exosomes can elicit functional responses such as remodelling the extracellular matrix and transmitting signals to recipient cells, and are involved in numerous physiological and pathological processes (Pegtel & Gould, 2019; Verweij et al., 2018). Exosomes secreted by tumour cells contribute to cancer initiation and development via influencing numerous neoplastic processes, including tumorigenesis, growth, metastasis, drug resistance and immune escape (L. Zhang & Yu, 2019; X. Zhang et al., 2015). Eliminating tumour‐derived exosomes by interfering with their biogenesis, transport, or secretion in tumour cells holds promise as a tumour treatment strategy (Bobrie et al., 2012; Chalmin et al., 2010).

Over the past few decades, significant efforts have been made to identify the biomolecules packaged within exosomes. However, the mechanisms responsible for MVB–PM fusion, the final step in exosome secretion, remain poorly understood. It is known that the core machinery controlling this step consists of SNAREs composed of one R‐SNARE and three Q‐SNAREs, which form the SNARE complex to provide energy for membrane fusion. To date, a number of SNAREs, such as Q_a_‐SNAREs syntaxin‐2 and syntaxin‐4 (Verweij et al., 2018; Y. Wang et al., 2021), Q_bc_‐SNARE SNAP‐23 (Verweij et al., 2018; Wei et al., 2017), Q_c_‐SNARE syntaxin‐6 (Peak et al., 2020), and R‐SNAREs Ykt6 and VAMP‐7 (Fader et al., 2009; Gross et al., 2012; Ruiz‐Martinez et al., 2016), have been implicated in exosome secretion in various organs hosing tumour cells. However, many challenges and controversies remain: (i) many of these investigations fail to consider the complexity of exocytic secretion pathways, leading to difficulties in discriminating between the necessity of SNAREs in MVB–PM fusion and the preceding MVB biogenesis or transportation processes; (ii) complete and cognate SNAREs required and specific for MVB–PM fusion have not been identified yet; and (iii) the potential of the SNAREs as therapeutic target for tumour treatment has not been tested in vivo. Consequently, there is a critical need for the identification of the SNARE complex composition that mediates MVB–PM fusion in exosome secretion, as well as a comprehensive evaluation of their expression, localization, and potency to mediate membrane fusion.

Breast cancer has become the most commonly diagnosed cancer globally, with an estimated burden of 2.3 million new cases in 2020 (H. Sung et al., 2021). In recent years, mounting studies have revealed the vital role of tumour‐derived exosomes in the pathophysiology of breast cancer (Deepak et al., 2020; Dong et al., 2020; Jabbari et al., 2020). Given the high incidence of breast cancer and the crucial role of tumour‐derived exosomes in breast cancer progression, in this study, we investigated the composition of the SNARE complex mediating MVB–PM fusion in MCF‐7 human breast cancer cells. Our findings indicate that Q_a_‐SNARE syntaxin‐4, Q_bc_‐SNARE SNAP‐23, and R‐SNARE VAMP‐7 are able to assemble into a SNARE complex that mediates MVB–PM fusion to accomplish exosome secretion in MCF‐7 cells. The same SNAREs were found to mediate exosome secretion in HeLa human cervical cancer cells and A375 human melanoma cancer cells. Furthermore, the deletion of VAMP‐7 in 4T1 murine mammary carcinoma cells significantly restrained the growth and metastasis of tumour in vivo. Taken together, these results for the first time identified the complete SNARE composition mediating MVB–PM fusion in MCF‐7 human breast cancer cells, and emphasize the potential of VAMP‐7 as a therapeutic target to suppress tumour‐derived exosome secretion, making this a promising strategy for the treatment of breast cancer.

## 2 MATERIALS AND METHODS

### 2.1 Antibodies and reagents

The following antibodies were used: Rabbit Polyclonal anti‐GFP (50430‐2‐AP, Proteintech), Rabbit Polyclonal anti‐mCherry (26765‐1‐AP, Proteintech), Mouse Monoclonal anti‐beta Actin (60008‐1‐Ig, Proteintech), Mouse Monoclonal anti‐SNAP‐25 (60159‐1‐Ig, Proteintech), Rabbit Polyclonal anti‐SNAP‐23 (10825‐1‐AP, Proteintech), Mouse Monoclonal anti‐SNAP‐29 (sc‐390602, Santa Cruz Biotechnology), Rabbit Monoclonal anti‐Syntaxin‐1A (A19243, ABclonal Technology), Rabbit Monoclonal anti‐Syntaxin‐4 (A5996, ABclonal Technology), Rabbit Polyclonal anti‐Syntaxin‐11 (13301‐1‐AP, Proteintech), Rabbit Monoclonal anti‐VAMP2 (A4235, ABclonal Technology), Mouse Monoclonal anti‐VAMP‐7 (232011, Synaptic Systems), Rabbit Polyclonal anti‐VAMP‐8 (15546‐1‐AP, Proteintech), Mouse Monoclonal anti‐Alix (sc‐53540, Santa Cruz Biotechnology), Mouse Monoclonal anti‐CD63 (sc‐5275, Santa Cruz Biotechnology), Mouse Monoclonal anti‐CD81 (sc‐166029, Santa Cruz Biotechnology), Mouse Monoclonal anti‐Flag (AE005, ABclonal Technology), HRP‐conjugated Affinipure Goat Anti‐Rabbit IgG(H+L) (SA00001‐2, Proteintech), HRP‐conjugated Affinipure Goat Anti‐Mouse IgG(H+L) (SA00001‐1, Proteintech), Goat anti‐Mouse IgG (H+L) Cross‐Adsorbed Secondary Antibody, Alexa Fluor™ 647 (A‐21235, Invitrogen).

100 μm histamine (S20188‐1 g, Shanghai yuanye Bio‐Technology Co., Ltd) was added to enhance the activity of exosome secretion. 100 μm leupeptin (SG2012‐10 mM, Beyotime) was applied to block MVB degradation.

### 2.2 Cell culture

HEK293T cells (ATCC), MCF‐7 cells (ATCC), HeLa cells (ATCC), A375 cells (ATCC) were cultured in DMEM medium (Gibco) with 10% FBS (Gibco) and 1% penicillin‐streptomycin (Biosharp). 4T1 cells (ATCC) were cultured in RPMI‐1640 medium (Gibco) supplemented with 10% FBS (Gibco) and 1% penicillin‐streptomycin (Biosharp). All cells were cultured in a humidified incubator at 37°C with 5% CO2.

### 2.3 CRISPR‐Cas9 and small guide RNAs (sgRNAs)

To generate SNARE knockout cell lines, CRISPR‐Cas9 system was used. HEK293T cells were transfected with lentiCRIPSR v2, where sgRNA targeting corresponding SNARE was inserted, psPAX2 and pMD2.G plasmids using Lipofectamine™ 3000 Transfection Reagent (L3000015, Invitrogen™) to generate lentivirus. After 36–48 h of transfection, the cell medium of HEK293T cells was collected and centrifuged to remove the cell debris. The supernatant containing lentivirus‐sgRNA was used to infect MCF‐7, HeLa, A375 and 4T1 cells. After 48 h of infection, 5–7 days of puromycin (P8032, Solarbio) selection at a concentration of 2.5 μg/mL was performed. Limiting dilution was applied to obtain sub‐cell line clone from MCF‐7, HeLa, A375 and 4T1 cells. Cells infected with lentivirus generated from HEK239T cells co‐transfected with empty lentiCRIPSR v2, psPAX2 and pMD2.G plasmids were as control. Western blot was used to substantiate the corresponding SNARE protein was successfully knocked out.

The sgRNAs used to knockout the proteins are as follows:

Syntaxin‐4: GGCAGACTATTGTCAAACTG, Syntaxin‐11: TGAGGCGCCGCATGGACGTG, SNAP‐23: GGTCCAAGCCTTCTTCTATG, SNAP‐29: GCATTAAGAGCGTGTTTGGG, VAMP‐7: CTATCCTTGCCAAACATGCT, VAMP‐2: GGAGCGAGACCAGAAGCTGT, VAMP‐8: CTTCAAGACGACATCGCAGA.

### 2.4 Plasmids, transfections, and protein purification

mOrange sequence was amplified out of NPY‐td‐Orange2 (Addgene, Plasmid #83497) and inserted into the first extracellular loop of CD63 between amino acids Gln36 and Leu37. The sequence of mOrange‐inserted CD63 was constructed into NPY‐td‐Orange2 vector. The sequences of cytoplasmic domain of human Syx‐2 (residues 190−260), Syx‐3 (residues 190−260), Syx‐4 (residues 199−269), Syx‐5 (residues 262−332), Syx‐7 (residues 164−234), Syx‐11 (residues 203−273), Syx‐12 (residues 177−274), Syx‐16 (residues 229−299), Syx‐17 (residues 161−231), Syx‐18 (residues 242−311), Syx‐19 (residues 208−278) were respectively cloned into pEGFP‐N3 vector (Clontech). The sequences of full‐length human Syx‐3 (residues 1−289), Syx‐4 (residues 1−297), Syx‐11 (residues 1−287), Syx‐18 (residues 1−335), VAMP‐2 (residues 1−116), VAMP‐3 (residues 1−100), VAMP‐4 (residues 1−141), VAMP‐7 (residues 1−220), VAMP‐8 (residues 1−100), Ykt6 (residues 1−198), SN‐23 (residues 1−211) were respectively constructed into pEGFP‐C1 vector (Clontech). The sequences of full‐length human Syx‐3, Syx‐4, Syx‐11, Syx‐18, SN‐23, SN‐25 (residues 1−300), SN‐29 (residues 1−258), SN‐47 (residues 1−464), Vti1A (residues 1−271), Vti1B (residues 1−232), GosR1 (residues 1−250), GosR2 (residues 1−212), Sec20 (residues 1−228), Syx‐6 (residues 1−255), Syx‐8 (residues 1−236), Syx‐10 (residues 1−249), Bet1 (residues 1−118), Bet1L (residues 1−111), Use1 (residues 1−259) were respectively constructed into pmCherry‐C1 vector (Clontech). The sequence of full‐length human Syx‐4 was constructed into pcDNA3.1‐ vector (Invitrogen) with an N‐terminal Flag‐tag. Transfection of plasmids mentioned above was performed using ExFect® Transfection Reagent (Vazyme) according to the manufacturer's protocol. 24–36 h after transfection, cells were ready for the next experiments. The sequences of full‐length human Syx‐4, the cytoplasmic fragment of Syx‐4 (residues 1–274), the mutant of human VAMP‐7 (Δ209−220, residues 1–208, Y45E, I139S, I144S, i.e., VAMP‐7 Δ12‐3 M) and the mutant of full‐length human SN‐23 (C79A, C80A, C83A, C85A, C87A, C112A) were respectively cloned into pET28a vector (Novagen) for protein purification. The sequence of human VAMP‐7 SNARE motif (residues 125–185) was constructed into pGEX‐6p‐1 vector (GE Healthcare) incorporating an N‐terminal PreScission protease‐cleavable glutathione‐S‐transferase (GST)‐tag for protein purification. These proteins were all expressed in Escherichia coli BL21 DE3 and purified as previously described. Briefly, cells were cultured in Luria‐Bertani (LB) medium and induced with 0.2 mM IPTG at OD_600_ of 0.6 for 18 h at 20°C. Cell pellets from 1.5 L of LB medium were resuspended with 50 mM Tris‐Cl, pH 8.0, 300 mM NaCl, 0.5% Triton X‐100 (Sigma, termed lysis buffer) supplied with 1 mM PMSF and 0.5 mM Tris(carboxyethyl)‐phosphine (TCEP), lysed with AH‐1500 Nano Homogenize Machine at 1200 bar for at 4°C, and centrifuged at 16,000 rpm in a JA‐25.50 rotor (Beckman Coulter) at 4°C. For hexa‐histidine‐tagged protein, the supernatants were incubated with 1 mL Nickel‐NTA agarose (Qiagen) affinity medium at 4°C for 2 h. After incubation, the mixture was washed twice with lysis buffer supplied with 0.5 mM TCEP and 30 mM imidazole. Transmembrane proteins Syx‐4 and VAMP‐7 were eluted with the elution buffer containing 20 mM Tris‐Cl, pH 8.0, 150 mM NaCl, 300 mM imidazole, 0.2 mM TCEP, and 1% (w/v) sodium cholate hydrate (Aladdin). SN‐23 was eluted by the elution buffer excluding 1% (w/v) sodium cholate hydrate. For GST‐tagged VAMP‐7 SNARE motif, the supernatants were collected and mixed with 1 mL glutathione Sepharose 4B (GE Healthcare) affinity media. After 4 h rotation at 4°C, the mixture was washed twice with the buffer containing 50 mM Tris‐Cl, pH 8.0, 300 mM NaCl. Lastly, the GST‐tagged protein used for pulldown assay, was eluted with the buffer containing 50 mM Tris‐Cl, pH 8.0, 300 mM NaCl and 10 mM glutathione (Biosharp).

### 2.5 TIRF detection of MVB‐PM fusion

Exosome secretion from live cells was monitored by TIRF microscopy (Nikon) as described previously (Bebelman et al., 2020; Verweij et al., 2018). Briefly, MCF‐7 cells were plated on NEST glass bottom dishes and transfected with CD63‐mOrnge and indicated pEGFP‐N3‐Q_a_‐SNARE cytoplasmic domain plasmids. After 24−48 h of transfection, cells were imaged by TIRF illumination on a Nikon Ti inverted microscope equipped with a 100× oil‐immerse objective (NA 1.49) and an EMCCD camera (Andor DU897). 100 μm histamine was added prior to the detection. EGFP fluorescence was excited by halogen lamp with GFP filter cube to ensure that the detecting cells successfully overexpressed the cytoplasmic fragment of Q_a_‐SNARE. Orange fluorescence was excited with a 532 nm laser at 2 Hz. Exocytosis events were scored manually and analyzed by ImageJ and Prism 6.0 software.

### 2.6 Fluorescence imaging

To analyze the colocalization between Q_a_‐SNARE and Q_bc_‐SNARE, MCF‐7 cells were plated on NEST glass bottom dishes and transfected with indicated pEGFP‐C1‐Q_a_‐SNARE plasmid and pmCherry‐C1‐Q_bc_‐SNARE plasmid. After 24–36 h of transfection, cells were fixed with 4% paraformaldehyde. To analyze the colocalization of R‐SNARE and CD63, MCF‐7 cells were transfected with pEGFP‐C1‐R‐SNARE plasmid and CD63‐mOrange plasmid followed by paraformaldehyde fixation, or after transfected with pEGFP‐C1‐R‐SNARE plasmid, MCF‐7 cells were fixed and stained with the anti‐CD63 primary antibodies (1:200) followed by Goat anti‐Mouse IgG (H+L) Cross‐Adsorbed Secondary Antibody, Alexa Fluor™ 647 (1:500). Cells were imaged by Nikon confocal microscopy equipped with a 60× oil‐immerse objective (NA 1.40), and the Pearson’ correlation was determined by NIS elements AR 4.40 and data analysis was acquired by Prism 6.0.

For quantification of the quantities of R‐SNAREs that overlapped with CD63 in Figure 4, Manders’ colocalization coefficients (MCC) (Manders et al., 1993) were applied. For fluorescence of EGFP‐tagged R‐SNAREs (*G*) and mOrange‐tagged or Alexa647‐immunostained CD63 (*R*), one of the MCC values that calculates the fraction of R‐SNAREs (*G*) in compartments containing CD63 (*G*) is given as:

$$\;\;\;\;\;\;\;M_{GtoR}=\frac{\sum_{i}G_{i,colocal}}{\sum_{i}G_{i}}\;$$

$$\left\{\begin{array}{c} \;G_{i,colocal}=G_{i}\;\;\left(R_{i}>0\right)\\ \;G_{i,colocal}=\;0\;\left(\;R_{i}=\;0\right) \end{array}\right.$$

Where the ‘CD63‐positive area’ (*R_i_* > 0) and ‘R‐SNARE‐positive area’ (*G_i_* > 0) were determined by using self‐adaptive Otsu thresholding algorithm (Otsu, 1979).

To quantify the number of MVBs in control, Syx‐4, SN‐23 and VAMP‐7 knockout MCF‐7 cells, cells were fixed with 4% paraformaldehyde and immunolabelled with anti‐CD63 primary antibodies (1:200) and Goat anti‐Mouse IgG (H+L) Cross‐Adsorbed Secondary Antibody, Alexa Fluor™ 647 (1:500). Confocal (60× objective) and TIRF microscopy (100× objective) were performed to image the cytoplasmic and peri‐membranal CD63^+^ puncta respectively, and the number of MVBs was calculated manually and analyzed by Prism 6.0.

### 2.7 FLIM‐FRET measurement

To assess the interplay between Q_a_‐SNAREs and Q_bc_‐SNAREs by FLIM‐FRET, MCF‐7 cells were transfected with pEGFP‐C1‐Q_a_‐SNARE plasmid and pmCherry‐C1 empty vector or pmCherry‐C1‐Q_bc_‐SNARE plasmid. FLIM imaging was performed using a Nikon C2+ microscope equipped with a 60× oil‐immerse objective (NA 1.40) and a time‐correlated single‐photon counting (TCSPC) module from PicoQuant. EGFP fluorescence was excited by a 485 nm picosecond laser diode (LDH‐D‐C‐485, PicoQuant) at 5 mW, 40 MHz using PDL 800‐D laser driver (PicoQuant), and the emission signal was collected through a 530/10 nm bandpass filter using a gated PMA hybrid photomultiplier (PMT) detector and a TimeHarp 260 TCSPC module (PicoQuant). SymPhoTime 64 software (PicoQuant) was used to fit fluorescence decay data from full images after reconvolution for the instrument response function.

### 2.8 Western blot

Cells or small extracellular vesicels (sEVs) were lysed in RIPA buffer (50 mM Tris‐HCl pH 7.5, 150 mM NaCl, 1% Triton X‐100) containing protease inhibitor cocktail (Topscience). Proteins were separated by 12% acrylamide/bisacrylamide gel electrophoresis, transferred to PVDF membranes (Millipore) and probed with indicated primary antibody followed by horseradish peroxidase (HRP)‐conjugated secondary antibody. Immunodetection was carried out with the super sensitive ECL luminescence reagent (MA0186‐2, meilunbio). The integrated density of blot strips was analyzed by ImageJ and Prism 6.0 software to characterize the relative protein level.

### 2.9 Co‐immunoprecipitation (co‐IP)

To explore the interactions among Syx‐4, SN‐23 and VAMP‐7, MCF‐7 cells were transfected with the indicative tag‐fused plasmids and lysed with RIPA buffer (50 mM Tris‐HCl pH 7.5, 150 mM NaCl, 1% Triton X‐100) containing protease inhibitor cocktail (Topscience). The lysates were cleared by centrifugation at 12,000 × g for 10 min at 4°C, in which the GFP‐tagged proteins were immunoprecipitated with anti‐GFP magnetic beads (L‐1016, biolinkedin) for 6 h at 4°C according to the manufacturer's instructions. Then the magnetic beads were washed three times with phosphate‐buffered saline (PBS), followed by addition of loading buffer, and samples were boiled at 95°C for 10 min for immunoblotting analysis.

### 2.10 GST pull‐down assay

To detect the interactions of VAMP‐7, Syx‐4 and SN‐23, 2 μM GST‐VAMP‐7 SNARE motif, together with 3 μM Syx‐4 (residues 1–274, the cytoplasmic fragment) and/or 3 μM SN‐23, was mixed with 20 μL 50% (v/v) glutathione Sepharose 4B affinity media (GE Healthcare) to a final volume of 100 μL at 4°C for 5 h. 2 μM GST protein with 3 μM Syx‐4 (residues 1–274, the cytoplasmic fragment) and 3 μM SN‐23, was mixed with 20 μL 50% (v/v) glutathione Sepharose 4B affinity media (GE Healthcare) to a final volume of 100 μL at 4°C for 5 h as control. The media‐bound samples were washed by washing buffer containing 20 mM Tris‐HCl, pH 8.0, 150 mM NaCl and 1% Triton X‐100 (Sigma) for three times, and the samples were boiled and analyzed by SDS‐PAGE.

### 2.11 Lipid‐mixing assay

1‐palmitoyl‐2‐oleoyl‐glycero‐3‐phosphocholine (POPC), 1‐palmitoyl‐2‐oleoyl‐sn‐glycero‐3‐phosphoethanolamine (POPE), and 1,2‐dioleoyl‐sn‐glycero‐3‐phospho‐(1′‐myo‐inositol‐3′‐phosphate) ammonium salt (PI[3]P) were obtained from Avanti Polar Lipids and dissolved in chloroform at a final concentration of 10 mg▪mL^‐1^ except for PI[3]P, which was dissolved in a mixture of chloroform:methanol 2:1 at a concentration of 1 mg▪ml‐1. 1,1′‐dioctadecyl‐3,3,3′,3′‐tetramethylindocarbocyanine (DiIC18(3)) and 1,1′′‐dioctadecyl‐3,3, 3′′,3′′‐tetramethylindodicarbocyanine (DiDC18(5)) were obtained from Molecular Probes and dissolved in ethanol at a concentration of 1 mg▪ml‐1. Lipids were mixed at the proper ratio as indicated below to a final concentration of 1 mM. Donor liposome (reconstituted with VAMP‐7 Δ12‐3 M) contains 75.5 % POPC, 20 % POPE, 3 % PI[3]P, and 1.5 % DiI (molar ratio). Acceptor liposome (reconstituted with full‐length Syx‐4) contains 75.5 % POPC, 20 % POPE, 3 % PI[3]P, and 1.5 % DiD (molar ratio). Lipid mixtures were dried under nitrogen flow and further incubating in vacuum for 1 h at room temperature in the dark. Lipid films were resuspended in buffer T (20 mM Tris‐Cl pH 8.0, 150 mM NaCl) supplied with 0.2 mM Tris (2‐Tris (2‐carboxyethyl) phosphine (TCEP, Sigma‐Aldrich) and 1 % CHAPS (w/v, Amresco). Purified proteins were added to resuspended lipids with a protein‐to‐lipid ratio of 1:500. After incubation on the ice for 20 min, lipid‐protein mixtures were desalted using PD‐10 desalting column (GE Healthcare). Prepared proteoliposomes were stored at 4°C in the dark before using. Liposome fusion assays were carried out using FluoDia T70 fluorescence plate reader (PTI) equipped with 530/10 excitation filter, 580/10 and 667/10 emission filters at 37°C. Donor and acceptor liposomes were mixed at a concentration of 100 μM (total lipids) with addition of 2 μM recombinant SN‐23. Donor (DiI) and acceptor (DiD) fluorescence were monitored every 25 seconds. It is inevitable that the fluorescence of DiI and DiD would undergo photobleach‐driven decay as a function of time during long‐term excitation. To better illustrate the liposome fusion data, corrections for donor/acceptor photobleaching have been made by monitoring the fluorescence of donor liposome (DiI) and acceptor liposome (DiD), respectively, as a function of time using identical experimental setup as in liposome fusion assays. Liposome fusion signals were interpreted as the FRET proximity ratio (*E*_PR_) (S. Wang & Ma, 2022) between the donor (DiI) and acceptor (DiD):

$$\;E_{PR}=\frac{I_{DiD}}{I_{DiD}+I_{DiI}}\;$$

Where the $I_{DiD}$ and $I_{DiI}$ are the corrected fluorescence intensities of DiD and DiI under the 530/10 excitation filter, respectively. All the experiments were independently repeated for three times.

### 2.12 sEVs isolation, nanoparticle tracking analysis and transmission electron microscopy

To isolate sEVs from cell medium, the equivalent number of control or SNARE knockout cells were plated on the 15‐cm culture dish. After cell adherence, cells were rinsed with PBS and refreshed with the equivalent volume of DMEM containing 10% sEVs‐depleted FBS (bovine sEVs were removed by overnight centrifugation at 100,000 × g) followed by a 24‐h incubation. To isolate sEVs from cell medium, sequential centrifugation was performed. Briefly, the medium was centrifuged at 300 × g for 10 min to remove cells, then centrifuged at 2000 × g for 20 min to remove cell debris, and next centrifuged at 10,000 × g for 30 min to remove large extracellular vesicles. The resulting supernatant was ultracentrifuged at 100,000 × g (Beckman Type 70Ti) for 70 min. The sEVs pellet was washed in cold PBS and collected by ultracentrifugation again at 100,000 × g (Beckman Type 70 Ti) for 70 min. Finally, the sEVs pellet was resuspended in PBS or RIPA buffer before further analysis.

The number and size of sEVs was directly detected by the NS300 instrument (Malvern) equipped with a high‐sensitivity sCMOS camera. Particles were automatically tracked and sized based on Brownian motion and the diffusion coefficient. Three 60‐s videos were acquired for each sample. The videos were subsequently analyzed with the NanoSight NTA software, which identified and tracked the center of each particle under Brownian motion to measure the average distance the particles moved on a frame‐by‐frame basis.

The morphology of sEVs was characterized by transmission electron microscopy. A 10‐μL aliquot of freshly isolated sEVs was dried on a formvar/carbon‐coated copper grid for 10 min and 10 μL 2% (w/v) phosphotungstic acid hydrate (P100467‐25 g, aladdin) was used to negatively stain the sample for 3 min at room temperature. The samples were then observed at 100 kV with a HITACHI transmission electron microscope (TEM).

The ILV formation within MVBs in control, Syx‐4, SN‐23 or VAMP‐7 knockout MCF‐7 cells was assessed by transmission electron microscopy. Cells were fixed in 2.5% glutaraldehyde overnight at 4°C. Then, samples were washed three times in PBS followed by fixation in 1% osmium tetroxide for 60 min at room temperature. After that, samples were washed three times in PBS and stained in 2% uranium acetate for 30 min. Following staining, samples were dehydrated by a graded ethanol series (50%, 70%, 90% and 100%) and 100% acetone, 15 min for each step. Next, samples were embedded in a 1:1 solution of epon:acetone for 2 h, followed by embedding in a 3:1 solution of epon:acetone overnight at room temperature. Subsequently, samples were placed in fresh epon for 8 h at 37°C. Following this, the samples were embedded in epon for a duration of 48 h at 60°C. Thin sections were cut with an ultramicrotome, collected on grids, and subjected to examination using an electron microscope (Hitachi, 80 kV).

### 2.13 Cell counting Kit‐8 (CCK‐8)

The proliferation of control and VAMP‐7 knockout 4T1 cells was assessed by CCK‐8 (A311‐01, Vazyme) according to the manufacturer's instructions. Briefly, cell suspension was added on 96‐well cell culture plates, and the viability of cells at the time point of 0, 24 h and 48 h was detected by CCK‐8 Kit.

### 2.14 In vivo assays

BALB/c mice (female, 5‐7‐week‐old) were purchased from Beijing Vital River Laboratory Animal Technology Co., Ltd. (Beijing, China). Mice were housed in an animal facility under constant environmental conditions (room temperature, 22  ±  1 °C; relative humidity, 40–70%; a 12 h light‐dark cycle). All mice had access to food and water ad libitum. All animal experiments were performed under the guidance and approved by the Institutional Animal Care and Use Committee at Tongji Medical College, Huazhong University of Science and Technology (Wuhan, China).

The orthotopic mouse models of breast cancer were established by inoculating control or VAMP‐7 knockout 4T1 cells (3 × 10^5^ cells per mouse) into the right forth breast fat pad of BALB/c female mice, and tumours were measured every other day from the 6^th^ day of inoculation with a digital caliper. The sizes of tumours were calculated as the following formula: (width)^2^ × length × 0.5. On the 30^th^ day of inoculation, mice were euthanized and blood samples of these mice were immediately collected in anticoagulant tubes respectively by cardiac puncture. 100‐μL serum samples were acquired by centrifuging blood samples at 3000 × rpm for 10 min. In case that the number of sEVs isolated from one serum sample was under the limit of detection, we mixed two serum samples as one test sample randomly followed by adding 20 mL PBS. Serum sEVs were isolated by sequential centrifugation as mentioned above, and were quantified by NTA. Tumours were dissected and weighted after euthanasia of the mice.

The lung metastasis models were established by intravenously injecting 1 × 10^5^ control or VAMP‐7 knockout 4T1 cells via tail vein. On the 25^th^ day of cell injection, the mice were sacrificed. The lungs were collected and fixed in Bouin's solution (Solarbio, Beijing, China) overnight at room temperature, and the metastatic nodules in the lungs were counted. Lastly, the lungs were sectioned and stain with H&E.

To examine the effect of sEVs on the oncogenicity and the lung metastasis competence of 4T1 cells, 1 × 10^9^ sEVs isolated from control 4T1 cells were injected into the indicated mouse via tail vein every other day as shown in Figure 9a,e.

### 2.15 RESULTS

#### 2.15.1 Screen of potential Qa‐SNAREs that mediate exosome secretion

**FIGURE 1:**
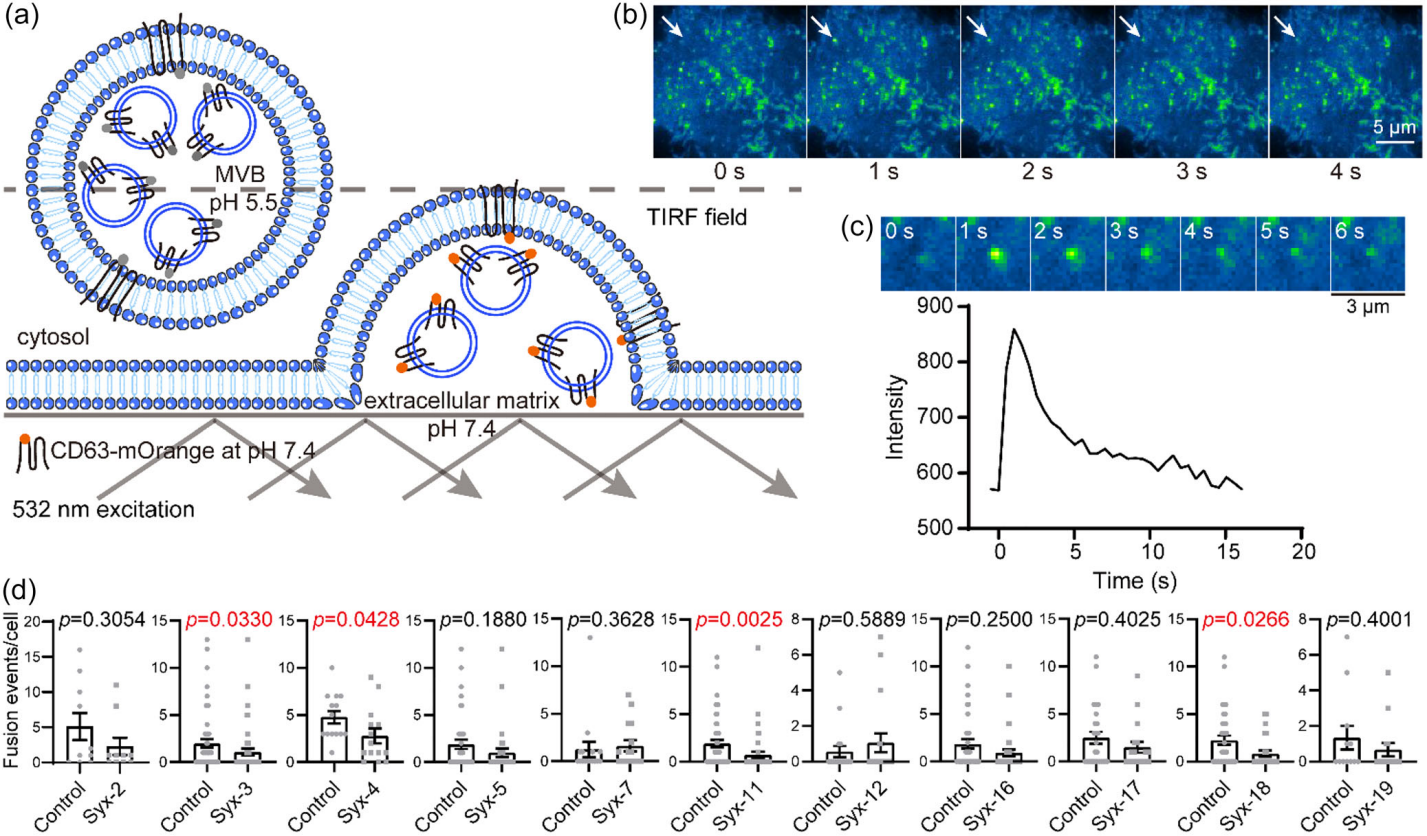
Screen of potential Qa‐SNAREs that mediate exosome secretion. (a) Illustration of the detection of MVB–PM fusion by TIRF microscopy. The fluorescent intensity of the pH‐sensitive optical reporter (CD63‐mOrange) significantly increases when ILVs (exosomes) are secreted from the acidic lumen of MVBs to the neutral extracellular matrix. (b) Representative live‐cell TIRF microscopy images, the white arrow indicates an MVB–PM fusion event. Scale bar, 5 μm. (c) Top, the enlarged ROI images of a representative MVB–PM fusion event. Bottom, representative fluorescence intensity (CD63‐mOrange) time course of a single MVB–PM fusion event. Scale bar, 3 μm. (d) Effects of overexpressing the cytoplasmic fragments of Qa‐SNAREs on the fusion activity of MCF‐7 cells. Control, empty vector transfected. Duration of TIRF imaging was 7 min for each cell. Cells that overexpressed a specific Qa‐SNARE mutant with an EGFP tag and their corresponding control cells were obtained from the same batch (culture dish), underwent identical experimental procedures, and were imaged in one same TIRF experiment. Data are presented as the means ± SEM, n ≥ 10 cells per condition from three separate replicates, Mann Whitney test was used for data analysis.

CD63, a member of tetraspanin family, is widely used as a marker protein to track exosomes in cells (Mathieu et al., 2021; B. H. Sung et al., 2020; Verweij et al., 2018). In order to visualize exosome secretion, a pH‐sensitive mOrange fluorescent reporter was inserted into the first extracellular loop of CD63 (termed CD63‐mOrange) and total internal reflection fluorescence (TIRF) microscopy was used to detect the fluorescent signal of mOrange near the PM (Figure 1a) (Gandasi et al., 2015; Verweij et al., 2018). Once the ILV (exosomes) within acidic MVBs were secreted to the extracellular matrix, the sudden increase of environmental pH led to a rapid and strong enhancement in the fluorescent signal of CD63‐mOrange (Figure 1a), which was followed by a rapid, exponential decrease in the fluorescence intensity lasting around 2 seconds at the fixed position (Figure 1b,c; Figure S1A and B; and Video S1). It is important to note that some vesicle activities that accompanied the increase in fluorescence intensity could not be attributed to MVB–PM fusion based on the the fluorescence profile (Figure S1A and B). For example, in kiss‐and‐run‐like events where there was a temporary opening and closure of small fusion pores between MVBs and the PM, there was an increase in the fluorescence intensity of CD63‐mOrange but without complete exosome secretion. This differed from MVB–PM fusion, where the fluorescent signal decayed rapidly upon fusion at the site of the fluorescent burst. In kiss‐and‐run‐like events, the increase in fluorescence intensity was followed by a delayed and irregular decrease along with lateral movement of the fluorescent signal (Figure S1A and B). Additionally, entry of transport vesicles carrying CD63‐mOrange with neutral pH into the TIRF field (without fusion with the PM) resulted in an increase in the fluorescent signal of CD63‐mOrange, with constant vesicle movement generating volatile fluorescence intensity and evident fluorescent shift (Figure S1A and B) (Bebelman et al., 2020). Using this approach, we analyzed exosome secretion in MCF‐7.

Overexpression of soluble mutant forms (i.e., the cytoplasmic fragments) of the SNAREs in cells is predicted to compete with the endogenous counterparts in the formation of SNARE complexes, thus inhibiting membrane fusion mediated by the endogenous SNAREs. Through this approach, potential Q_a_‐SNAREs that mediate exosome secretion in MCF‐7 cells were screened. Syntaxin‐1, which has a specific role in exocytosis of synaptic vesicles and dense‐core vesicles in neuronal and neuroendocrine cells (Burgoyne & Morgan, 2003; Jahn & Scheller, 2006), was excluded due to its expression being undetectable in MCF‐7 cells (Figure S2A). Of the remaining mammalian Q_a_‐SNAREs, including syntaxin‐2, −3, −4, −5, −7, −11, −12, −16, −17, −18, and −19, the mutant forms of each Q_a_‐SNAREs tagged with EGFP were co‐expressed with CD63‐mOrange in MCF‐7 cells (Figure S2B), and their influences on exosome secretion were monitored by TIRF microscopy as described in Figure 1a–c. To facilitate assessment of the effect of Q_a_‐SNARE mutants on MVB–PM fusion over a brief duration, 100 μM histamine was added to the cell culture medium prior to the detection (Bebelman et al., 2020; Verweij et al., 2018). Among the examined Q_a_‐SNAREs, the expression of soluble mutant forms of syntaxin‐3, −4, −11, and −18 (referred to as Syx‐3, −4, −11, and −18 hereafter), respectively, significantly inhibited exosome secretion (Figure 1d), which indicates that they might be potential Q_a_‐SNAREs involved in mediating exosome secretion.

#### 2.15.2 Search of potential Qbc‐SNAREs colocalizing with the Qa‐SNARE candidates

**FIGURE 2:**
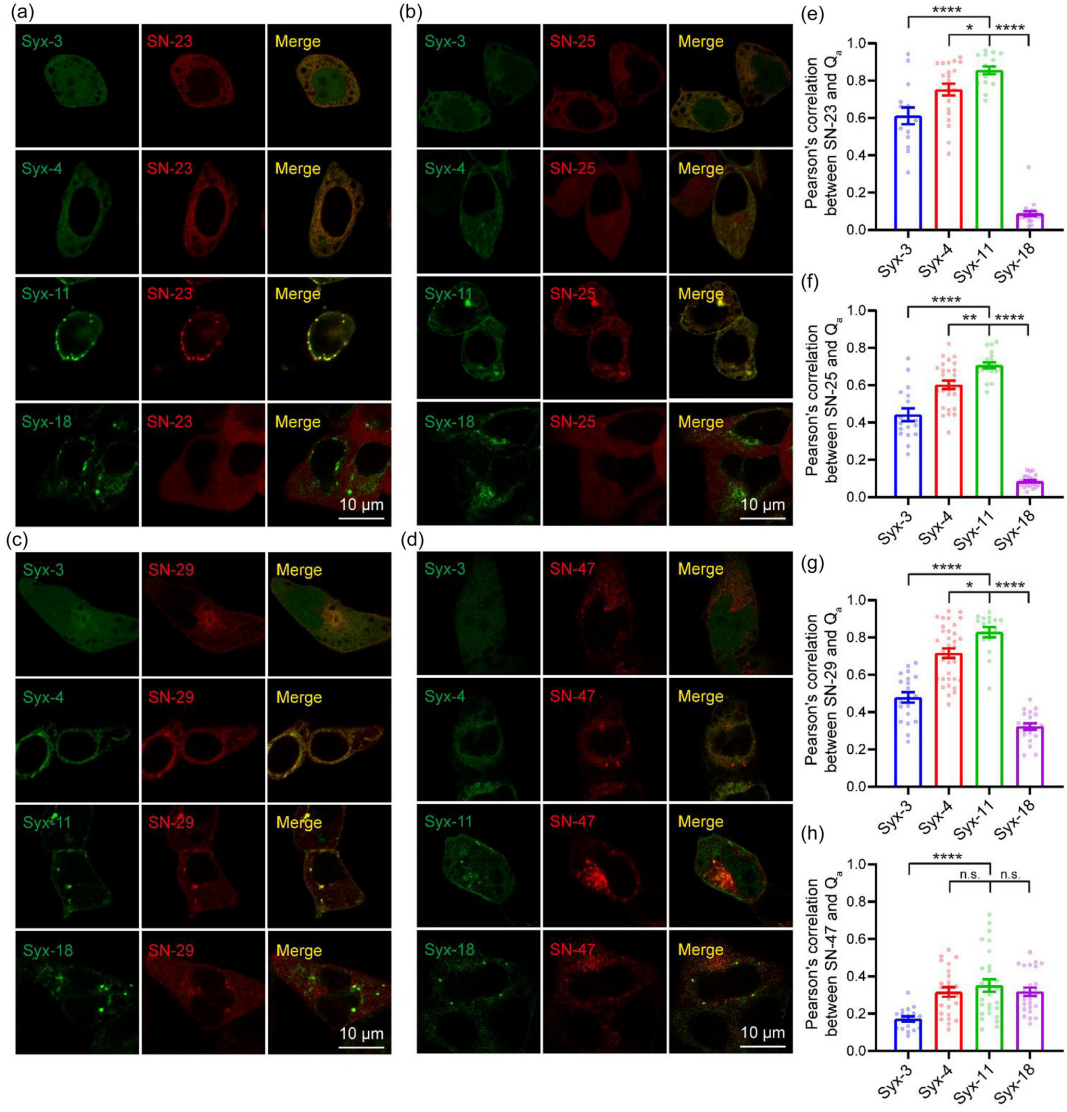
Search of potential Qbc‐SNAREs colocalizing with the Qa‐SNARE candidates. (a–d) Representative confocal images of MCF‐7 cells co‐expressing EGFP‐Syx‐3, −4, −11, or −18 and mCherry‐SN‐23 (a), −25 (b), −29 (c) and −47 (d), respectively. Scale bar, 10 μm. (e–h) Pearson's correlation between EGFP‐Syx‐3, −4, −11, or −18 and mCherry‐SN‐23 (e), −25 (f), −29 (g) or −47 (h) in MCF‐7 cells respectively. Data are presented as the means ± SEM, n ≥ 15 cells (e), 17 cells (f), 16 cells (g) or 18 cells (h) per condition from three separate replicates, ordinary one‐way ANOVA, *p < 0.05, **p < 0.01, ***p < 0.001, ****p < 0.0001, n.s., no significance.

Next, we sought to search potential Q_bc_‐SNAREs that have the same spatiotemporal location as the Q_a_‐SNARE candidates identified above in MCF‐7 cells. We tested four Q_bc_‐SNAREs, that is, SNAP‐23, −25, −29, and −47 (referred to as SN‐23, −25, −29, and −47 hereafter) known to mediate diverse membrane trafficking and fusion events in mammalian cells. A cross‐combination strategy was employed to co‐express each Q_bc_‐SNARE with the identified Q_a_‐SNARE candidates in MCF‐7 cells. Confocal microscopy was used to measure the colocalization between mCherry‐tagged Q_bc_‐SNARE and EGFP‐tagged Q_a_‐SNARE in each combination. We found that SN‐23, −25, and −29 exhibited significant colocalization with Syx‐3, −4, and −11, whereas limited colocalization was detected between Syx‐18 and these Q_bc_‐SNAREs (Figure 2a–c and e−g; and Table S1). This suggests that Syx‐3, −4, and −11 in combination with SN‐23, −25, and −29 may be the most likely candidates to compose the cognate Q_a_‐SNAREs and Q_bc_‐SNAREs that mediate exosome secretion. In contrast, SN‐47 failed to colocalize with the examined Q_a_‐SNAREs (Figure 2d,h; and Table S1), implying that SN‐47 is unlikely to be involved in exosome secretion. Notably, the expression of SN‐25 was undetectable in MCF‐7 cells (Figure S3), so it was ruled out as a potential Q_bc_‐SNARE. Hence, we selected SN‐23 and −29 as the potential Q_bc_‐SNAREs for further investigation based on the colocalization data.

### 2.16 Analysis of interactions between the Q_a_‐SNAREs and Q_bc_‐SNAREs

**FIGURE 3:**
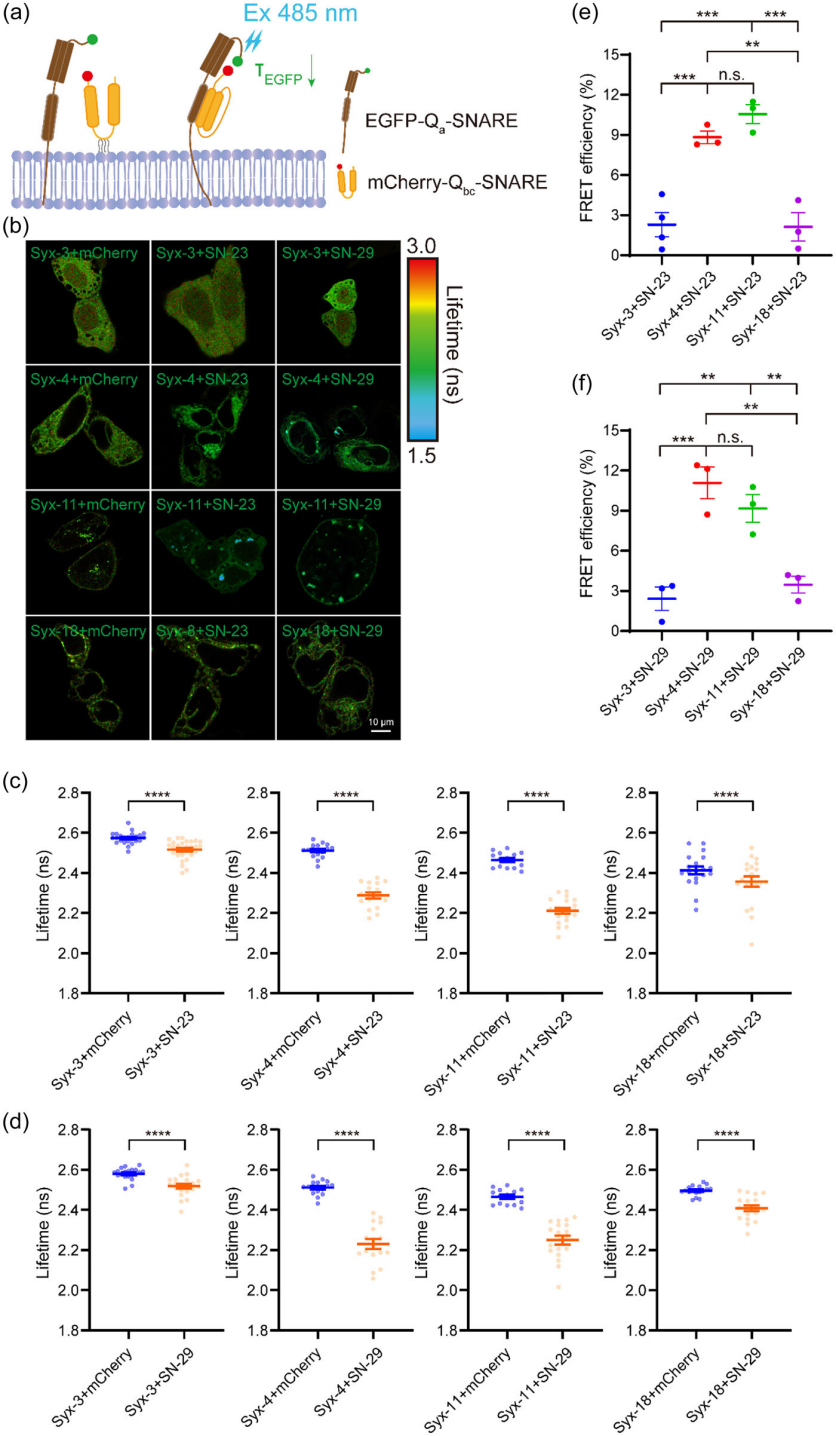
Analysis of interactions between the Qa‐SNAREs and Qbc‐SNAREs. (a) Illustration of FLIM‐FRET applied for detecting the interactions between the Qa SNAREs and Qbc‐SNAREs. FRET occurs when the donor and acceptor are in close physical proximity, leading to a decrease in fluorescence lifetime of the EGFP‐Qa‐SNARE protein. (b) FLIM images of MCF‐7 cells co‐expressing EGFP‐Syx‐3, −4, −11, or −18 and mCherry, mCherry‐SN‐23 or −29, respectively. Scale bar, 10 μm. (c) Averaged lifetime of EGFP‐Syx‐3, −4, −11 or −18 in the presence of mCherry or mCherry‐SN‐23 in MCF‐7 cells. (d) Averaged lifetime of EGFP‐Syx‐3, −4, −11 or −18 in the presence of mCherry or mCherry‐SN‐29 in MCF‐7 cells. Data are presented as the means ± SEM, n ≥ 14 cells per condition from four independent replicates, two‐tailed t test, ****p < 0.0001 (c and d). (e and f) FRET efficiency between EGFP‐Syx‐3, −4, −11 or −18 and mCherry‐SN‐23 (e) or −29 (f) in MCF‐7 cells. FRET efficiency in % = (1 − (τ DA/τ D)) × 100, τ DA represents the averaged lifetime of EGFP‐tagged protein (donor, D) in the presence of the mCherry‐tagged protein (acceptor, A) and τ D represents the averaged lifetime of an EGFP‐tagged protein (donor, D) in the presence of the mCherry. Data are presented as the means ± SEM, n = 4 independent replicates, ordinary one‐way ANOVA, **p < 0.01, ***p < 0.001, n.s., no significance (e and f).

Colocalization is not always a reliable indicator of interactions in vivo. To obtain independent information about distances and the fraction of interacting and non‐interacting proteins, we used fluorescence lifetime imaging microscopy & fluorescence resonance energy transfer (FLIM‐FRET), which measures the change in the fluorescence lifetime (τ) decay of the FRET donor (Becker, 2012; Wallrabe & Periasamy, 2005). We generated EGFP‐tagged Q_a_‐SNAREs (i.e., Syx‐3, −4, −11, −18) and mCherry‐tagged Q_bc_‐SNAREs (i.e., SN‐23 and −29) (Figure 3a), and used FLIM‐FRET to analyze their interactions by means of the same cross‐combination strategy as described in Figure 2. When donor and acceptor proteins are in close proximity (∼50 Å), FRET occurs resulting in a decrease in the fluorescence lifetime of EGFP‐Q_a_‐SNAREs (Figure 3a). We observed different levels of decrease in the fluorescence lifetime of EGFP‐Q_a_‐SNAREs when co‐expressed with mCherry‐SN‐23 or −29, with EGFP‐Syx‐4 or −11 showing more significant decreases than EGFP‐Syx‐3 or −18 (Figure 3b–d; Figure S4; and Table S2). Moreover, EGFP‐Syx‐4 or −11 produced higher FRET efficiency than EGFP‐Syx‐3 or −18 when co‐expressed with mCherry‐SN‐23 or −29 (Figure 3e,f; and Table S2). These data reveal the binding preference between Q_a_‐SNAREs Syx‐4 and −11 and Q_bc_‐SNAREs SN‐23 and −29 in MCF‐7 cells, giving us insight into the actual interactions between these proteins in vivo.

### 2.17 R‐SNARE VAMP‐7 colocalizes with the MVB marker CD63

**FIGURE 4:**
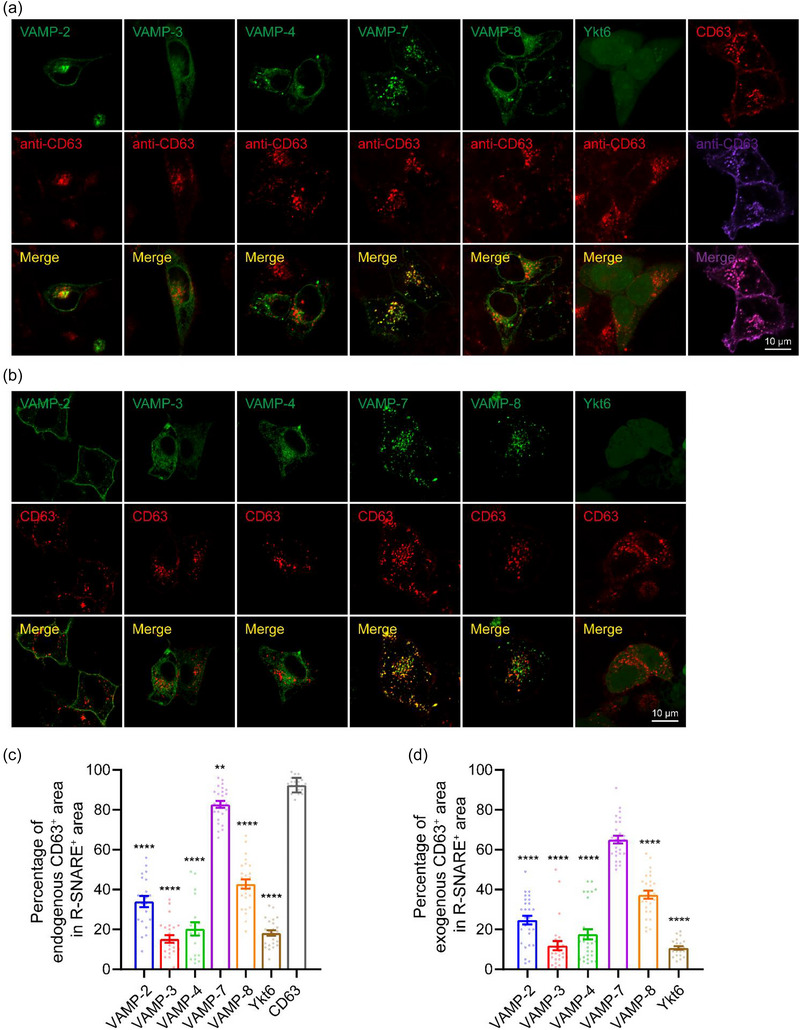
R‐SNARE VAMP‐7 colocalizes with the MVB marker CD63. (a) Representative confocal images of MCF‐7 cells expressing EGFP‐VAMP‐2, −3, −4, −7, −8, Ykt6 or CD63‐mOrange and immunolabeled with mouse anti‐CD63 primary antibody and Alexa Fluor™ 647‐conjugated goat anti‐mouse secondary antibody. (b) Representative confocal images of MCF‐7 cells co‐expressing EGFP‐VAMP‐2, −3, −4, −7, −8, or Ykt6 and CD63‐mOrange. Scale bar, 10 μm (a and b). (c and d) Percentage of EGFP‐VAMP‐2, −3, −4, −7, −8, Ykt6 or CD63‐mOrange positive area that is CD63 (endogenous in (c) or exogenous in (d)) positive. Data are presented as the means ± SEM, n ≥ 19 cells (c) or 22 cells (d) per condition from three separate replicates, ordinary one‐way ANOVA, **p < 0.01, ****p < 0.0001.

Exosome secretion requires the fusion of MVBs with the PM. The R‐SNAREs are typically embedded on the membrane of MVBs. To investigate which R‐SNAREs are involved in this fusion event leading to exosome secretion, we examined the colocalization of six R‐SNAREs (VAMP‐2, −3, −4, −7, −8, and Ykt6) with the MVB marker CD63 using confocal microscopy. We expressed each of the exogenous R‐SNAREs in EGFP‐tagged form individually in MCF‐7 cells, and visualized MVBs using endogenous CD63 immunostaining. VAMP‐7 showed the highest degree of colocalization with CD63, with up to 83% of EGFP‐positive puncta enriched on MVBs (Figure 4a,c; and Table S3). VAMP‐8 and VAMP‐2 exhibited partial colocalization with CD63, with around 42% and 34% of EGFP‐positive puncta enriched on MVBs, respectively (Figure 4a,c; and Table S3). On the other hand, VAMP‐3, VAMP‐4, and Ykt6 did not significantly colocalize with CD63, indicating their absence from MVBs (Figure 4a,c; and Table S3). These findings suggest that VAMP‐7, VAMP‐8, and VAMP‐2 are potentially involved in mediating the fusion of MVBs with the PM, and thus exosome secretion.

In addition, we substantiated the above results using exogenous co‐expression of EGFP‐tagged R‐SNAREs and mOrange‐tagged CD63 in MCF‐7 cells (Figure 4b,d; and Table S3). The exogenously expressed CD63‐mOrange exhibited punctate structures and subcellular distribution patterns similar to the endogenous CD63 (Figure 4a,b). Confocal imaging showed that VAMP‐7 exhibited the highest degree of colocalization with CD63‐mOrange puncta, while VAMP‐8 and −2 partially colocalized with CD63‐mOrange. However, VAMP‐3, VAMP‐4, and Ykt6 showed almost no colocalization with CD63‐mOrange (Figure 4b,d and Table S3). These findings indicate that VAMP‐7 is the most likely the R‐SNARE responsible for mediating exosome secretion, consistent with recent finding that VAMP‐7 decorates MVBs and participates in the fusion of MVB with the PM in human leukemic K562 cells (Fader et al., 2009).

### 2.18 Identification of syntaxin‐4, SNAP‐23 and VAMP‐7 as the cognate SNAREs that mediate MVB–PM fusion

**FIGURE 5:**
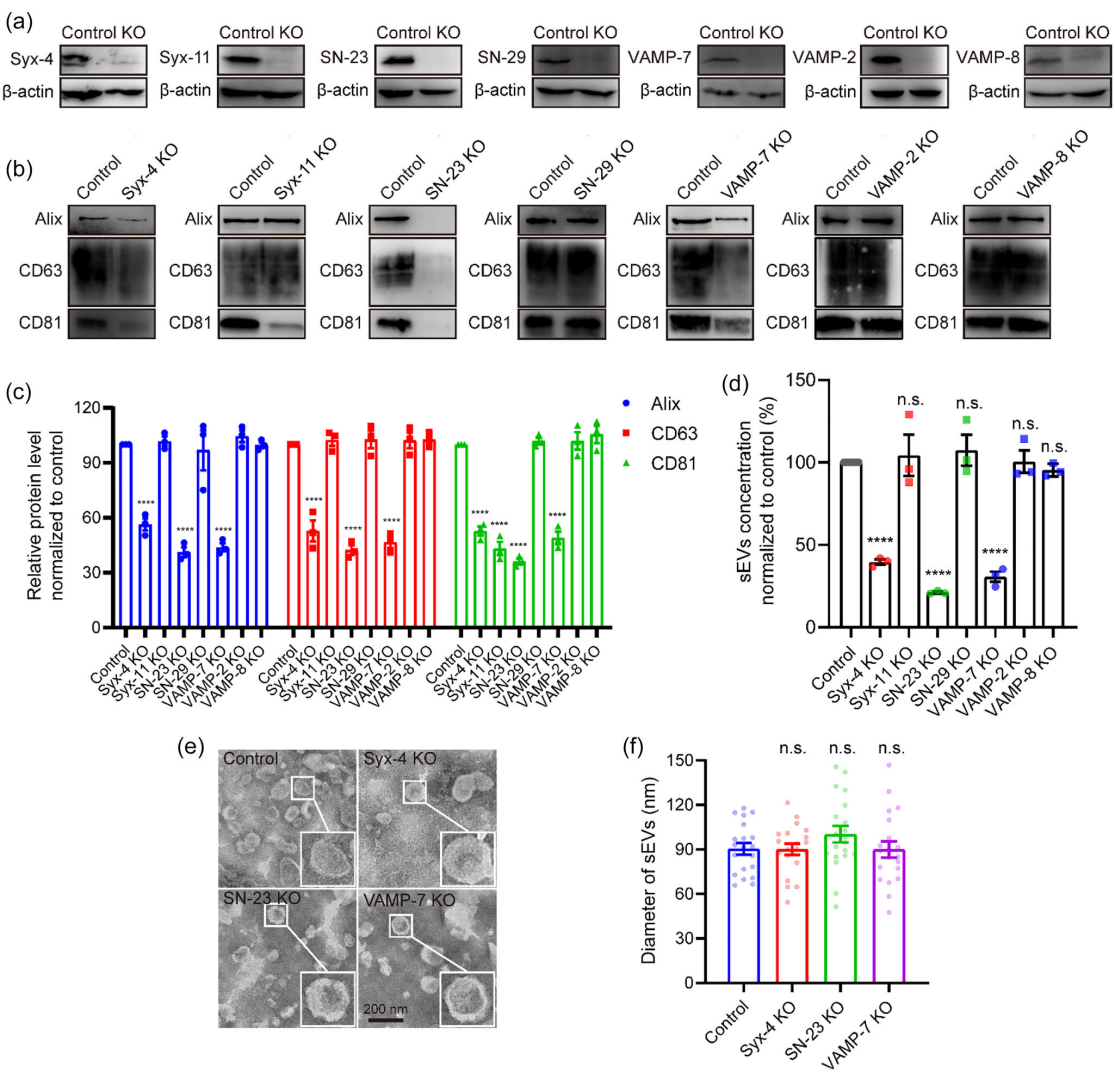
Identification of syntaxin‐4, SNAP‐23 and VAMP‐7 as the cognate SNAREs that mediate MVB‐PM fusion. (a) Validation of Syx‐4, Syx‐11, SN‐23, SN‐29, VAMP‐7, VAMP‐2 and VAMP‐8 knockout in MCF‐7 cells by western blot. (b) Western blot analysis of Alix, CD63 and CD81 in sEVs isolated from the same volume of cell culture supernatants of equal numbers of Syx‐4, Syx‐11, SN‐23, SN‐29, VAMP‐7, VAMP‐2 or VAMP‐8 knockout and control cells. (c) Relative protein level of Alix, CD63 and CD81 in the sEVs secreted from equivalent number of control and SNARE knockout MCF‐7 cells. Data are presented as the means ± SEM, n = 3 independent replicates, ordinary two‐way ANOVA, ****p < 0.0001. (d) Quantification of sEVs isolated from the same volume of cell culture supernatants of equal numbers of control and Syx‐4, Syx‐11, SN‐23, SN‐29, VAMP‐7, VAMP‐2 or VAMP‐8 knockout MCF‐7 cells by NTA. Data are presented as the means ± SEM, n = 3 independent replicates, ordinary one‐way ANOVA, ****p < 0.0001, n.s., no significance. (e) Representative TEM images of sEVs from control, Syx‐4, SN‐23 or VAMP‐7 knockout MCF‐7 cells. Scale bar, 200 nm. (f) Diameter of sEVs from control, Syx‐4, SN‐23 or VAMP‐7 knockout MCF‐7 cells by TEM. Data are presented as the means ± SEM, n = 20 sEVs, ordinary one‐way ANOVA, n.s., no significance.

To validate the roles of the Q_a_‐SNARE candidates Syx‐4 and −11, Q_bc_‐SNARE candidates SN‐23 and −29, and R‐SNARE candidate VAMP‐7, we individually knocked out these candidates in MCF‐7 cells by using CRISPR‐Cas9 system (Figure 5a). We also knocked out VAMP‐2 and VAMP‐8 in MCF‐7 due to their partial colocalization with CD63 (Figure 4a–d; and Table S3). Control cells infected with lentivirus carrying no sgRNA were used for comparison. Although there are observable disparities in the biogenesis of exosomes and other small extracellular vesicles (sEVs) such as ectosomes, discerning exosomes from ectosomes post cell‐secretion is experimentally challenging (Choi et al., 2015; Cocucci & Meldolesi, 2015). In our experiments, we isolated total sEVs containing exosomes from equal numbers of control and SNARE‐knockout cells using differential centrifugation approaches (Poggio et al., 2019; Thery et al., 2006) and analyzed the sEVs by immunoblotting of the exosome marker proteins Alix, CD63, and CD81 (Kowal et al., 2016; Mathieu et al., 2021; Verweij et al., 2018). Results showed that deletion of Syx‐4, SN‐23, or VAMP‐7 significantly reduced the amount of these marker proteins in the sEVs, while deletion of SN‐29, VAMP‐2, or VAMP‐8 did not (Figure 5b,c). It is notable that deletion of Syx‐11 specifically reduced CD81 rather than Alix and CD63 (Figure 5b,c), implying a role of Syx‐11 in the transporting CD81 into MVBs, particularly ILVs. Consistently, nanoparticle tracking analysis (NTA) showed that deletion of Syx‐4, SN‐23, or VAMP‐7 significantly decreased the amount of secreted sEVs (Figure 5d and Figure S5A). co‐IP experiment confirmed mutual interactions of exogenously expressed Syx‐4, SN‐23, and VAMP‐7 in MCF‐7 cells (Figure S5B). Hence, these data suggest that Syx‐4, SN‐23 and VAMP‐7 are required for exosome secretion.

**FIGURE 6:**
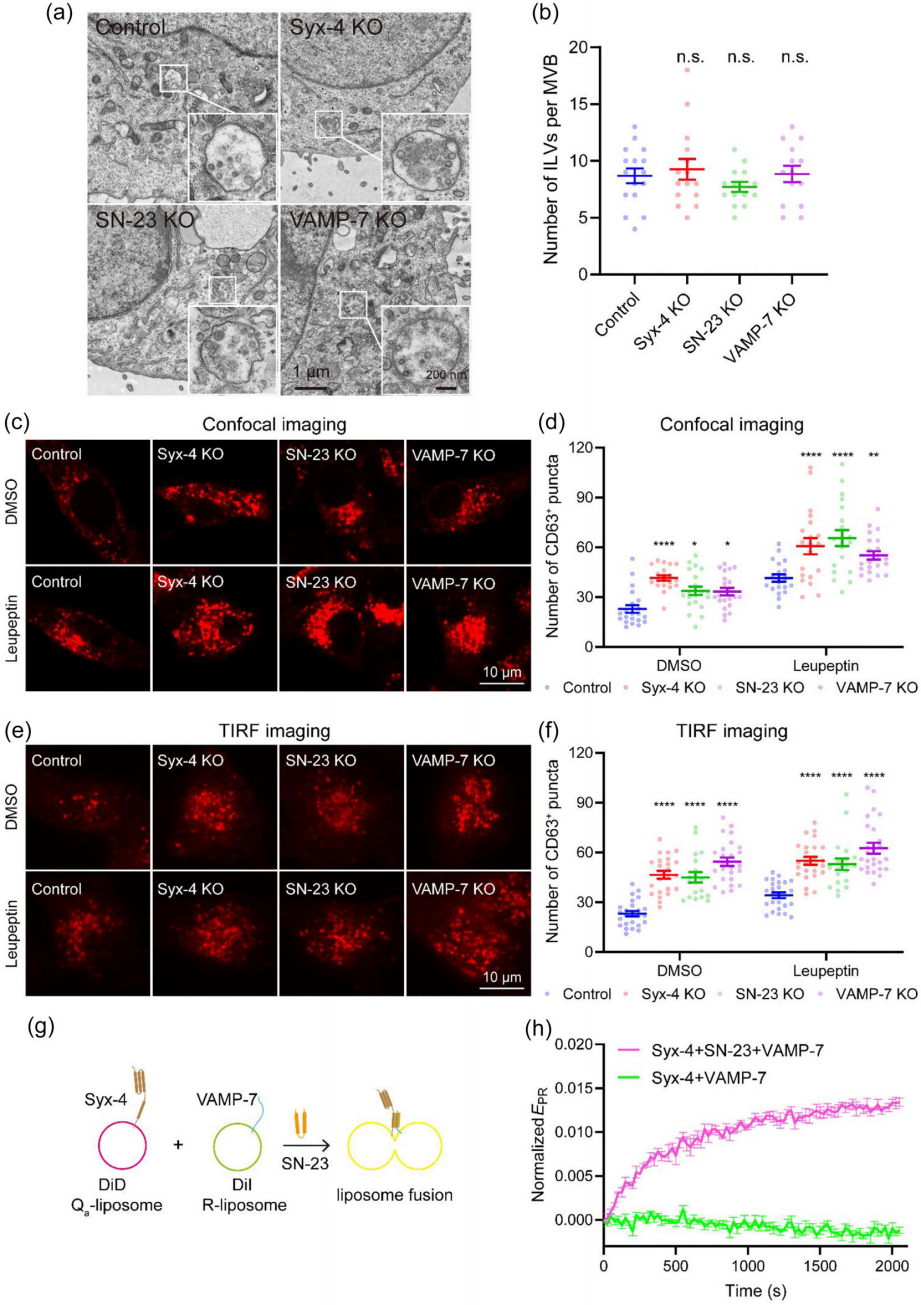
Confirmation of syntaxin‐4, SNAP‐23 and VAMP‐7 as the cognate SNAREs functioning in MVB–PM fusion. (a) Representative TEM images of MVBs in control, Syx‐4, SN‐23 or VAMP‐7 knockout MCF‐7 cells. Scale bar, 1 μm in wide filed and 200 nm in enlarged view. (b) Quantification of ILVs with MVBs in control, Syx‐4, SN‐23 or VAMP‐7 knockout MCF‐7 cells. Data are presented as the means ± SEM, n ≥ 14 MVBs, ordinary one‐way ANOVA, n.s., no significance. (c and e) Representative confocal (c) or TIRF (e) images of control, Syx‐4, SN‐23 or VAMP‐7 knockout MCF‐7 cells with (bottom) or without (top) 100 μm‐leupeptin treatment, immunolabeled with mouse anti‐CD63 primary antibody and Alexa Fluor™ 647‐conjugated goat anti‐mouse secondary antibody. Scale bar, 10 μm. (d and f) Quantification of the CD63+ puncta in MCF‐7 cells examined in (c) or (e), respectively. Data are presented as the means ± SEM, n ≥ 20 cells (c) or 19 cells (e) per condition from three separate replicates, ordinary two‐way ANOVA, *p < 0.05, **p < 0.01, ****p < 0.0001. (g) Illustration of liposome fusion assay. In the presence of SN‐23, the DiD labeled Syx‐4 liposomes fuse with the DiI labeled VAMP‐7 Δ12‐3 M liposomes, thus leading to FRET between two liposomes. (h) Time‐dependent liposome fusion measured from the development of FRET between the DiD labeled Syx‐4 liposomes and the DiI labeled VAMP‐7 Δ12‐3 M liposomes. Each trace is the average from three independent replicates. Error bars for each scatter indicate standard deviations (SD). Raw data for calculating EPR were corrected by filtering photobleach of donor and acceptor fluorescence.

Further investigation was carried out to confirm the function of Syx‐4, SN‐23 and VAMP‐7 in MVB–PM fusion. CD63‐mOrange was overexpressed in MCF‐7 cells lacking the corresponding SNAREs, and TIRF microscopy was used to investigate the influence of Syx‐4, SN‐23, and VAMP‐7 on MVB–PM fusion. Results showed that knockout of the three SNAREs inhibited MVB–PM fusion activity significantly (Figure S5C). To confirm the importance of Syx‐4, SN‐23, and VAMP‐7 in both basal and histamine‐stimulated exosome secretion, the quantification of sEVs secreted from cells treated with or without 100 μM histamine during a 24‐h incubation period was conducted by NTA. The administration of histamine augmented the quantity of sEVs derived from control, Syx‐4, and VAMP‐7 knockout MCF‐7 cells, but not the SN‐23‐deleted cells. Worth noting, the number of sEVs secreted from Syx‐4, SN‐23, or VAMP‐7 knockout cells was remarkably lower, regardless of the presence or absence of histamine treatment, compared to that from control cells (Figure S5D). This indicates that Syx‐4, SN‐23, and VAMP‐7 are indispensable for exosome secretion both on basal and histamine‐stimulated condition. In addition, the secreted sEVs from control and SNARE‐knockout cells displayed similar morphological characteristics of exosomes (Thery et al., 2006), with a diameter around 100 nm in average by TEM observation and NTA detection (Figure 5e,f; and Figure S5A), ruling out the influence of SNARE deletion on the morphology and the size distribution of the exosomes. Moreover, TEM analysis revealed that the knockout of Syx‐4, SN‐23, or VAMP‐7 had negligible impact on the formation of ILVs within MVBs (Figure 6a,b), suggesting that the reduced exosome secretion observed in SNARE‐knockout cells dues unlikely to inhibitions in ILV formation. In addition, we observed a remarkable accumulation of MVBs in SNARE‐knockout cells compared to control cells both in the absence and presence of protease inhibitor leupeptin (Figure 6c,d), a regent used to block MVB degradation (Aoyagi et al., 1969), indicating that knockout of Syx‐4, SN‐23, or VAMP‐7 is unlikely to affect MVB biogenesis. Finally, one may question whether the cytoplasmic stack of MVBs and the blockage of exosome secretion arise because deletion of Syx‐4, SN‐23, or VAMP‐7 influences the translocation of MVBs to the PM. To test this possibility, we tracked MVBs beneath the PM using TIRF microscopy. Indeed, more MVBs were detected in the subplasmalemmal field in Syx‐4, SN‐23, or VAMP‐7 deleted cells than in control cells in the absence and presence of leupeptin (Figure 6e,f), suggesting that the translocation of MVBs to the PM is not impaired in the SNARE‐deficient cells. Altogether, these data suggest that the deletion of Syx‐4, SN‐23, or VAMP‐7 specifically impairs MVB–PM fusion, but exerts no influence on MVB formation and translocation.

Apart from Q_bc_‐SNAREs, we explored whether Q_b_‐ or Q_c_‐SNAREs are involved in MVB–PM fusion in MCF‐7 cells. We analyzed the colocalization of Q_b_‐ (i.e., Vti1A, Vti1B, GosR1, GosR2 and Sec20) or Q_c_‐SNAREs (i.e., Syx‐6, Syx‐8, Syx‐10, Bet1, Bet1L and Use1) existing in mammalian cells with Syx‐4 (Figure S6A, B, D and E; and Table S4). Among them, the Q_b_‐SNARE Sec20 exhibited strong colocalization with Syx‐4 (Figure S6A and D; and Table S4), and the Q_c_‐SNARE Use1 mostly overlapped with Syx‐4 (Figure S6B and E; and Table S4). However, FLIM‐FRET analysis revealed that either mCherry‐SEC20 or mCherry‐Use1 showed no significant effect on the lifetime of EGFP‐Syx4 (Figure S6C and F; and Table S5), in contrast to the Q_bc_‐SNARE SN‐23 tagged with mCherry (Figure 3 and Table S2). These results indicate that Sec20 and Use1 are unlikely to interact with Syx‐4 despite strong subcellular colocalization. Sec20 and Use1 are primarily present in the endoplasmic reticulum (ER) and are involved in mediating retrograde vesicular transport from the Golgi apparatus to the ER (Meiringer et al., 2011), their colocalization with Syx‐4 might be important for the course of synthesis and processing of Syx‐4, rather than acting as the SNAREs for MVB–PM fusion.

We next conducted pull‐down and lipid‐mixing experiments to explore whether Syx‐4, SN‐23, and VAMP‐7 form a functional SNARE complex to drive MVB–PM fusion. As shown in Figure S5E, GST‐tagged VAMP‐7 SNARE motif (residues 125−185), Syx‐4 (residues 1−274, the cytoplasmic fragment), and SN‐23 successfully assembled into a 1:1:1 ternary complex, indicating their strong potency to form the SNARE complex. Then, we tested their ability to drive membrane fusion in vitro (Figure 6g). Since VAMP‐7 favors a closed conformation with its N‐terminal longin domain bound to its SNARE motif, we introduced Y45E/I139S/I144S mutation (3 M) to facilitate the opening of VAMP‐7 (Burgo et al., 2013; Fader et al., 2009; Schafer et al., 2012) and eliminated the C‐terminal 12‐amino acids to improve VAMP‐7 solubility. With lipid‐mixing assays, we observed significant membrane fusion between VAMP‐7 Δ12‐3 M liposomes and Syx‐4 liposomes in the presence of SN‐23 (Figure 6h and Figure S5F, G and H), demonstrating that the SNARE complex composed of Syx‐4, SN‐23, and VAMP‐7 is competent in mediating MVB‐PM fusion in MCF‐7 cells.

### 2.19 Syntaxin‐4, SNAP‐23 and VAMP‐7 mediate exosome secretion in HeLa and A375 cells

**FIGURE 7:**
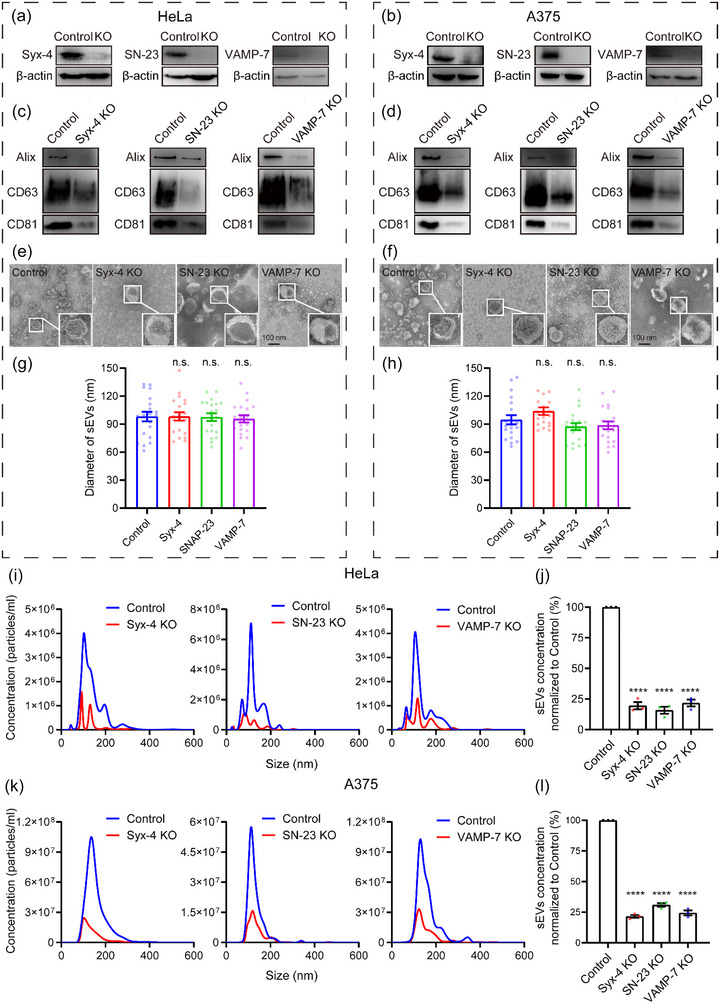
Syntaxin‐4, SNAP‐23 and VAMP‐7 mediate exosomes secretion in HeLa and A375 cells. (a and b) Validation of Syx‐4, SN‐23 and VAMP‐7 knockout in HeLa (a) and A375 (b) cells by western blot. (c and d) Western bolt analysis of Alix, CD63 and CD81 in sEVs isolated from the same volume of cell culture supernatants of equal numbers of control and Syx‐4, SN‐23 or VAMP‐7 knockout HeLa (c) or A375 (d) cells. (e and f) Representative TEM images of sEVs from control, Syx‐4, SN‐23 or VAMP‐7 knockout HeLa (e) or A375 (f) cells. (g and h) Diameter of sEVs from control, Syx‐4, SN‐23, or VAMP‐7 knockout Hela (g) or A375 (h) cells by TEM. Data are presented as the means ± SEM, n = 20 sEVs, ordinary one‐way ANOVA, n.s., no significance. (i and k) Representative NTA traces of sEVs from equal numbers of control and Syx‐4, SN‐23 or VAMP‐7 knockout HeLa (i) or A375 (k) cells. (j and l) Quantification of sEVs isolated from the same volume of cell culture supernatants of equal numbers of control and Syx‐4, SN‐23 or VAMP‐7 knockout HeLa (j) or A375 (l) cells by NTA. Data are presented as the means ± SEM, n = 3 independent replicates, ordinary one‐way ANOVA, ****p < 0.0001.

To explore whether Syx‐4, SN‐23 and VAMP‐7 mediate exosome secretion in other types of tumour cells, we generated individual knockout cell lines for each of these genes using CRISPR‐Cas9 in HeLa human cervical carcinoma cells and A375 human melanoma cells we individually knocked out Syx‐4, SN‐23 and VAMP‐7 in HeLa human cervical carcinoma cells and A375 human melanoma cells using CRISPR‐Cas9 system (Figure 7a,b). Similar to the observations in MCF‐7 cells, the knockout of Syx‐4, SN‐23, or VAMP‐7 significantly reduced the amount of secreted exosomes detected by immunoblotting of Alix, CD63, and CD81 (Figure 7c,d) as well as NTA analysis (Figure 7i–l). Moreover, the secreted sEVs from the knockout cells exhibited similar morphology and size distribution as those from control cells, as determined by TEM and NTA (Figure 7e–i,k). These data suggest that the roles of Syx‐4, SN‐23, and VAMP‐7 in mediating exosome secretion are critical and conserved in different types of tumour cells.

### 2.20 Inhibition of exosome secretion by deleting the SNAREs suppresses tumour progression

Exosomes are involved in local and distant cell communication, essential for numerous aspects of tumour progression such as growth, metastasis, drug resistance and immunoregulation (L. Zhang & Yu, 2019; X. Zhang et al., 2015). Given the importance of Syx‐4, SN‐23 and VAMP‐7 in exosome secretion in various tumour cells, it is expected that inhibition of exosome secretion by deleting the SNAREs would suppress tumour progression. To test this hypothesis, we used breast cancer as the tumour model and chose the R‐SNARE VAMP‐7 as the target protein. We generated a VAMP‐7‐deleted 4T1 murine mammary carcinoma cell line (Figure S7A) and confirmed that sEVs secretion was reduced (Figure S7D and E) without altering the sEVs’ morphology and size (Figure S7B–D) or affecting 4T1 cell proliferation (Figure S7F).

**FIGURE 8:**
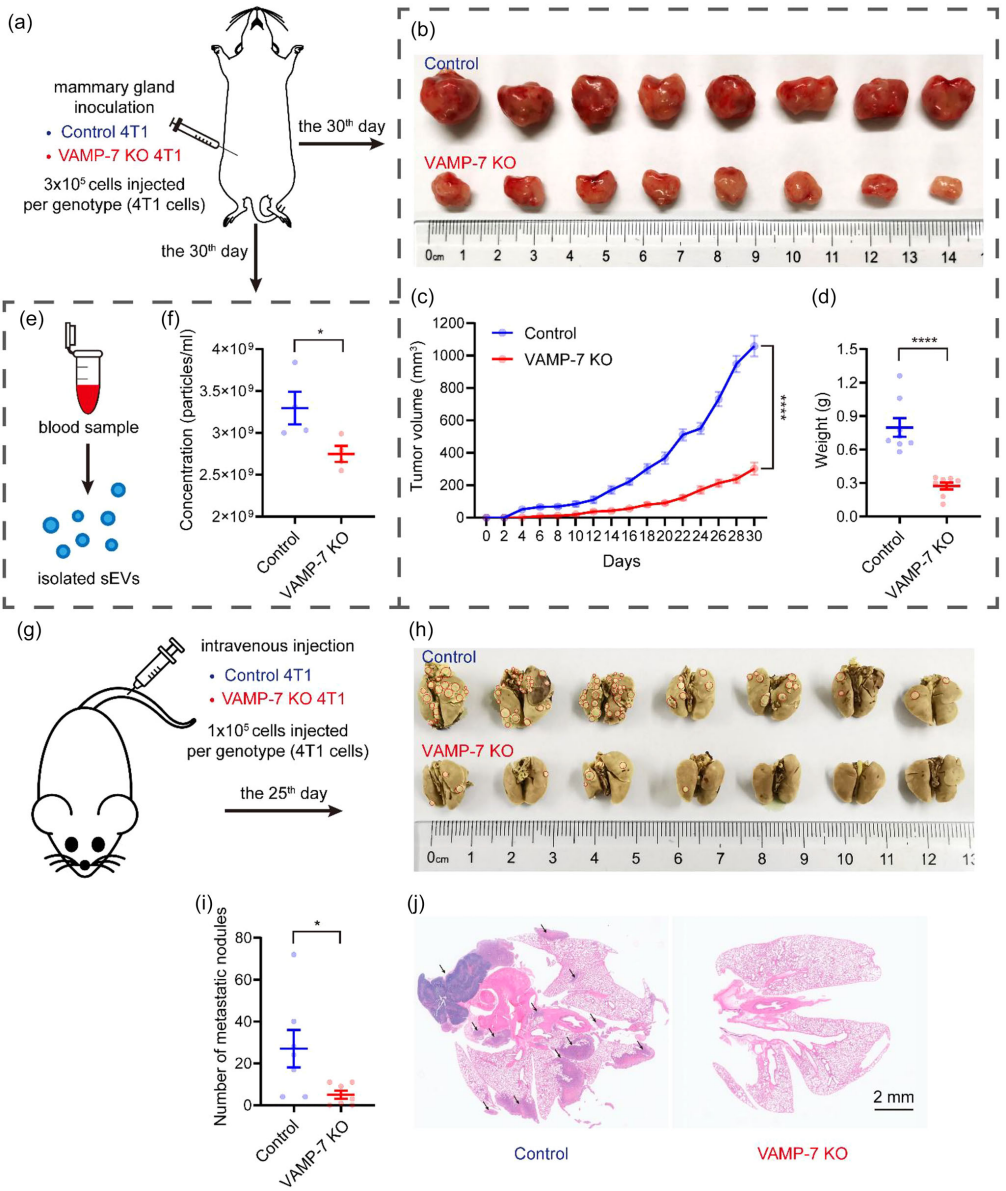
Inhibition of exosome secretion by deleting the SNAREs suppresses tumour progression. (a) schematic of experimental design for (b‐f). (b) Photograph of dissected tumours. Top, tumours dissected from mice inoculated with control 4T1 cells. Bottom, tumours dissected from mice inoculated with VAMP‐7 deleted 4T1 cells. (c) Tumour growth volume over time following mammary gland inoculation of control or VAMP‐7 knockout 4T1 cells. Data are presented as the means ± SEM, n = 8 mice for each genotype, two‐tailed t test, ****p < 0.0001. (d) Weight of dissected tumours shown in (b). Data are presented as the means ± SEM, n = 8 tumours for each group, two‐tailed t test, ****p < 0.0001. (e) Schematic of experimental design for (f). (f) Quantification of serum sEVs from tumour‐bearing mice inoculated with control or VAMP‐7 knockout 4T1 cells. Data are presented as the means ± SEM, n = 4 for each group, two‐tailed t test, *p < 0.05. (g) Schematic of experimental design for (h‐j). (h) Photograph of dissected lungs. Top, lungs dissected from mice injected with control 4T1 cells. Bottom, tumours dissected from mice injected with VAMP‐7 deleted 4T1 cells. Red circles are indicative of visualized metastatic nodules. (i) Quantification of metastatic nodules in lungs shown in (h). Data are presented as the means ± SEM, n = 7 mice for each genotype, two‐tailed t test, *p < 0.05. (j) Pathological examination of dissected lungs by H&E staining. Black arrows are indicative of metastasis areas in lung tissue.

To investigate whether VAMP7 knockout reduces the oncogenicity and the lung metastasis potential of 4T1 cells, we compared the competence of proliferation and metastasis between control and VAMP‐7 knockout cells in vivo. The orthotopic mouse models of breast cancer was established with control and VAMP7 knockout 4T1 cells (Figure 8a). The mice inoculated with VAMP‐7 knockout 4T1 cells exhibited a significant delay in tumour growth compared to those implanted with control 4T1 cells (Figure 8b–d). Generally, tumour cells secrete more exosomes than normal cell (Melo et al., 2014; Milane et al., 2015; Riches et al., 2014), and tumour‐derived exosomes (TDEs) can transmit carcinogenic cargoes distally through blood circulation. As expected, the quantity of circulating sEVs isolated from tumour‐bearing mice inoculated with VAMP‐7 knockout 4T1 cells was significantly reduced compared to those injected with control 4T1 cells (Figure 8e,f).

The majority of mortality from cancer is attributable to metastasis (Valastyan & Weinberg, 2011), and abundant evidence suggests that TDEs facilitate tumour metastasis (Mashouri et al., 2019). Hence, we explored whether the deletion of VAMP‐7 could suppress the lung metastasis potential of 4T1 cells in vivo. An experimental lung metastasis model was established by intravenous injecting control or VAMP‐7 knockout cells (Mohanty & Xu, 2010; Thies et al., 2020) (Figure 8g), and the lungs were dissected and evaluated for metastatic nodules when the mice were sacrificed. The numbers of macroscopic metastatic nodules were counted to assess the lung metastasis competence of control and VAMP‐7 knockout 4T1 cells. Mice injected with VAMP7‐deleted 4T1 cells displayed fewer lung metastatic foci than those injected with control 4T1 cells (Figure 8h,i). Additionally, lung sections stained with hematoxylin and eosin (H&E) exhibited histological differences between the two groups (Figure 8j), with the lungs of mice injected with VAMP‐7 knockout cells showing abundant sparse holes compared to the control group, which displayed multiple metastatic nodules arranged in tight alignments (Figure 8j). In conclusion, the lung metastasis competence of 4T1 cell was compromised on condition that VAMP7 was deleted.

**FIGURE 9:**
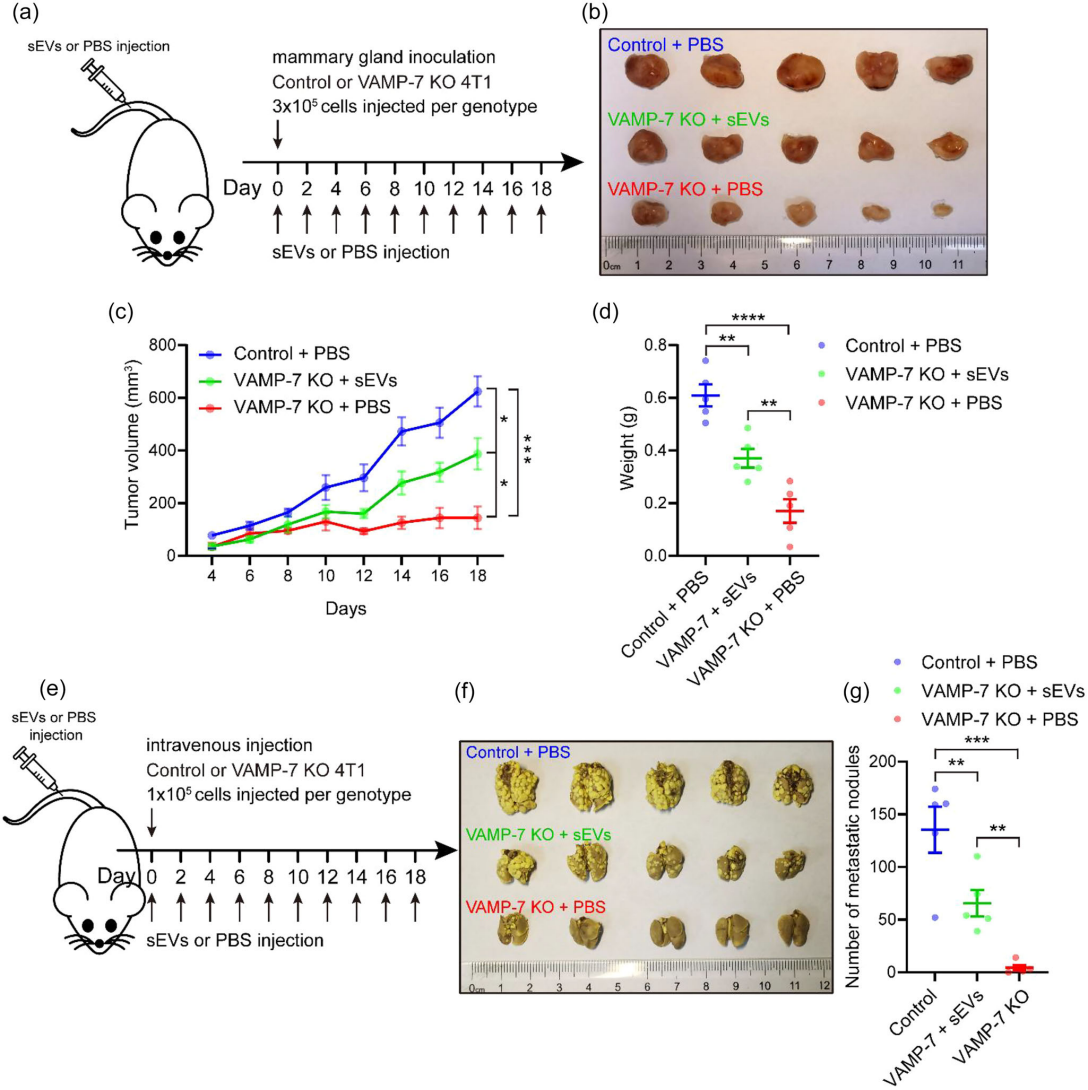
Validation that VAMP‐7 knockout restrained the oncogenicity and the lung metastasis competence of 4T1 cells by suppressing exosome secretion. (a) Schematic of experimental design for (b‐d). (b) Photograph of dissected tumours. Top, tumours dissected from mice inoculated with control 4T1 cells and injected with PBS. Middle, tumours dissected from mice inoculated with VAMP‐7 deleted 4T1 cells and injected with sEVs derived from control 4T1 cells. Bottom, tumours dissected from mice inoculated with VAMP‐7 deleted 4T1 cells and injected with PBS. (c) Tumour growth volume over time following mammary gland inoculation of control or VAMP‐7 knockout 4T1 cells and accompanying injection of indicated PBS or sEVs. Data are presented as the means ± SEM, n = 5 mice for each group, two‐tailed t test, *p < 0.05, ***p < 0.001. (d) Weight of dissected tumours shown in (b). Data are presented as the means ± SEM, n = 5 tumours for each group, two‐tailed t test, **p < 0.01, ****p < 0.0001. (e) Schematic of experimental design for (f and g). (f) Photograph of dissected lungs. Top, lungs dissected from mice injected with control 4T1 cells and PBS. Middle, lungs dissected from mice inoculated with VAMP‐7 deleted 4T1 cells and injected with sEVs derived from control 4T1 cells. Bottom, tumours dissected from mice injected with VAMP‐7 deleted 4T1 cells and PBS. (g) Quantification of metastatic nodules in lungs shown in (f). Data are presented as the means ± SEM, n = 5 mice for each genotype, two‐tailed t test, **p < 0.01, ***p < 0.001.

To confirm that the impaired oncogenicity and lung metastasis potential of VAMP‐7‐deficient 4T1 cells is due to the inhibition of exosome secretion, we established orthotopic or metastasis mouse models of breast cancer using control or VAMP‐7 knockout 4T1 cells, followed by injections of sEVs collected in vitro from control 4T1 cells or PBS (as a negative control) (Figure 9a,e). As shown in Figure 9b–d, treatment with sEVs significantly facilitated tumour growth in mice inoculated with VAMP‐7‐deleted 4T1 cells. Similarly, treatment with sEVs exhibited more lung metastatic nodules in mice injected with VAMP‐7 knockout cells (Figure 9f,g). It is important to note that slower tumour growth and fewer metastatic nodules were observed in mice inoculated with VAMP‐7‐deficient cells and treated with sEVs compared to those transplanted with control 4T1 cells and treated with PBS (Figure 9b–d,f and g). This observation may be due to the clearance of sEVs in vivo and limited sEVs accumulation in the tumour microenvironment through blood circulation. Overall, these findings confirm the crucial role of sEVs in tumour development and the critical nature of VAMP‐7 in tumour‐derived exosome secretion.

## 3 DISCUSSION

Tumour cells are able to adapt to the microenvironment thus contributing to tumour survival and progression via secreting exosomes and releasing heterogeneous exosomal cargoes. However, the specific SNARE complex mediating exosome secretion in tumour cells remains unknown. In this study, we identified syntaxin‐4, SNAP‐23, and VAMP‐7 as the cognate SNAREs that mediate exosome secretion in MCF‐7 human breast cancer cells. Moreover, we demonstrated that these SNAREs formed a functional complex capable of driving membrane fusion in vitro. Our data provided evidence that deletion of syntaxin‐4, SNAP‐23, or VAMP‐7 had no significant influence on MVB formation and translocation, but strongly impaired MVB–PM fusion leading to reduced exosome secretion in MCF‐7 cells. Importantly, syntaxin‐4, SNAP‐23, and VAMP‐7 were also found to mediate exosome secretion in other types of tumour cell lines, for example, HeLa and A375 cells, suggesting their functional importance and conservation in exosome secretion across diverse tumour types.

A number of SNAREs have been previously implicated in exosome secretion in different tumour cells, yet the exact components forming the SNARE complex that mediate MVB–PM fusion and exosome secretion in tumour cells has not been explicitly defined. For instance, it was reported that downregulation of syntaxin‐4 and SNAP‐23 in HeLa cells inhibits MVB–PM fusion (Verweij et al., 2018), and knockdown of SNAP‐23 in A549 human non‐small‐cell lung carcinoma cells reduces exosome secretion (Wei et al., 2017). Our results support these findings, as we identified syntaxin‐4 and SNAP‐23 as the Q_a_‐SNARE and Q_bc_‐SNARE responsible for exosome secretion in MCF‐7, HeLa, and A375 cells. In addition, other studies found that VAMP‐7, but not VAMP‐3, is essential for exosome secretion in K562 human chronic myeloid leukemia cells (Fader et al., 2009), which aligns with our identification of VAMP‐7 as the R‐SNARE that mediates exosome secretion in tumour cells. These findings suggest that syntaxin‐4, SNAP‐23, and VAMP‐7 are likely the key components of the SNARE complex that mediate exosome secretion in tumour cells.

In addition to the SNAREs described above, some other SNAREs have been mentioned to participate in exosome secretion. For example, upregulation of the Q_a_‐SNARE syntaxin‐2 was observed to promote exosome secretion in SW480 human colorectal cancer cells (Y. Wang et al., 2021). However, this study did not test whether syntaxin‐2 deletion reduces exosome secretion or whether syntaxin‐2 specifically mediates MVB‐PM fusion. Likewise, downregulation of the Q_c_‐SNARE syntaxin‐6 suppressed exosome secretion in enzalutamide‐resistant prostate cancer (PCa) cells (Peak et al., 2020), but did not provide evidence of the impact of syntaxin‐6 knockdown on exosome secretion directly. Thus, whether syntaxin‐6 participates in the constitutive secretion of exosomes and whether it functions in MVB–PM fusion remain elusive. Another study showed that knockdown of R‐SNARE Ykt6 inhibits exosome secretion in A549 human non‐small‐cell lung carcinoma cells (Ruiz‐Martinez et al., 2016). Again, this work did not provide direct evidence that Ykt6 mediates MVB–PM fusion, leaving open the possibility that these SNAREs interfere with upstream steps in the exosome secretion pathway, such as MVB generation and translocation. Despite that different tumour cells may use different SNAREs to mediate exosome secretion, further investigation is needed to determine the functional importance of syntaxin‐2, syntaxin‐6, and Ykt6 in exosome secretion in different tumour cells.

In neurons, neurotransmitter release by synaptic exocytosis is mediated by the SNARE complex composed of syntaxin‐1, SNAP‐25 and VAMP‐2 and many SNARE regulatory components such as Munc13‐1, Munc18‐1, synaptotagmin‐1, *etc* (Jahn & Fasshauer, 2012). Likewise, exosome secretion by MVB–PM fusion is accomplished by the coordination between the SNAREs and their regulators. Interestingly, more recent work found that Ca^2+^‐stimulated exosome secretion in MDA‐MB‐231 human breast cancer cells is regulated by Munc13‐4 (Messenger et al., 2018). Future investigations need to identify key components that regulate the assembly of syntaxin‐4, SNAP‐23, and VAMP‐7 and explore the underlying regulatory mechanisms.

In addition to mediating exosome secretion in multiple types of tumour cells, syntaxin‐4, SNAP‐23, and VAMP‐7 have been implicated in diverse membrane fusion events, for example, (i) Ca^2+^‐triggered exocytosis of conventional lysosomes in NRK rat kidney cells (Rao et al., 2004); (ii) transportation of membrane type 1‐matrix metalloproteinase (MT1‐MMP) to invadopodia involved in ECM degradation and tumour cell invasion (Williams et al., 2014); and (iii) exocytosis of typhoid toxin upon infection of *Salmonella* Typhi in HEK293T cells (Chang et al., 2022). In general, the functional importance of syntaxin‐4, SNAP‐23, and VAMP‐7 in membrane fusion may be attributed to their ubiquitous expression pattern across diverse cell types (Chen & Scheller, 2001). It needs to be emphasized that the above‐described membrane trafficking events typically belong to exocytic secretion pathways; this is not surprising because syntaxin‐4 and SNAP‐23 are predominantly found in the PM (Ren et al., 2017), and VAMP‐7 localizes to late endosomes and lysosomes (Tian et al., 2021), which might account for their functional specificity in exocytic secretion events.

Exosomes have been increasingly implicated in breast cancer progression (Jabbari et al., 2020; Joyce et al., 2016). Growing attempts have been made to suppress tumour development via inhibiting exosome secretion by genetic manipulation or application of exosome biogenesis inhibitor. Previous studies have found that inhibition of exosome secretion by deletion of exosome secretion regulator Rab27a or NSMASE2, or treatment with exosome secretion inhibitor GW4869, significantly restrain tumour growth and augment the efficacy of immunotherapy in vivo (Poggio et al., 2019; Yang et al., 2018). In our study, we established a tumour‐bearing mice model and found that breast tumour growth was suppressed and the quantity of serous sEVs was reduced in the tumour‐bearing mice inoculated with VAMP7‐deleted 4T1 cells. In addition, lung metastasis experiments demonstrated that VAMP‐7 knockout suppressed the lung metastatic competence of 4T1 cells. Further sEVs injection assays confirmed the significant contribution of sEVs to tumour development and the requirement of VAMP‐7 for tumour‐derived exosome secretion. Altogether, our findings, combined with previous research, demonstrate that blocking exosome secretion is a promising strategy for tumour treatment, with SNAREs mediating exosome secretion being ideal targets for tumour treatment.

## AUTHOR CONTRIBUTIONS

Chuqi Liu and Dexiang Liu performed most of the experiments; Shen Wang performed lipid‐mixing assay; Chuqi Liu, Dexiang Liu, and Shen Wang performed data analysis; Lu Gan, and Xiangliang Yang helped with in vivo experiments; Cong Ma and Chuqi Liu wrote the manuscript; Cong Ma conceived the project.

## CONFLICTS OF INTEREST

The authors declare no conflicts of interest.

## Supporting information

Supporting Information

Supporting Information
