# Supplementary material for: Identification of the SNARE complex that mediates the fusion of multivesicular bodies with the plasma membrane in exosome secretion
Source: J Extracell Vesicles. 2023 Sep 12;12(9):12356. doi: 10.1002/jev2.12356 (PMC10497535; doi:10.1002/jev2.12356)
Supplement: Supplementary file 1 — Supporting Information [file JEV2-12-12356-s001.docx]

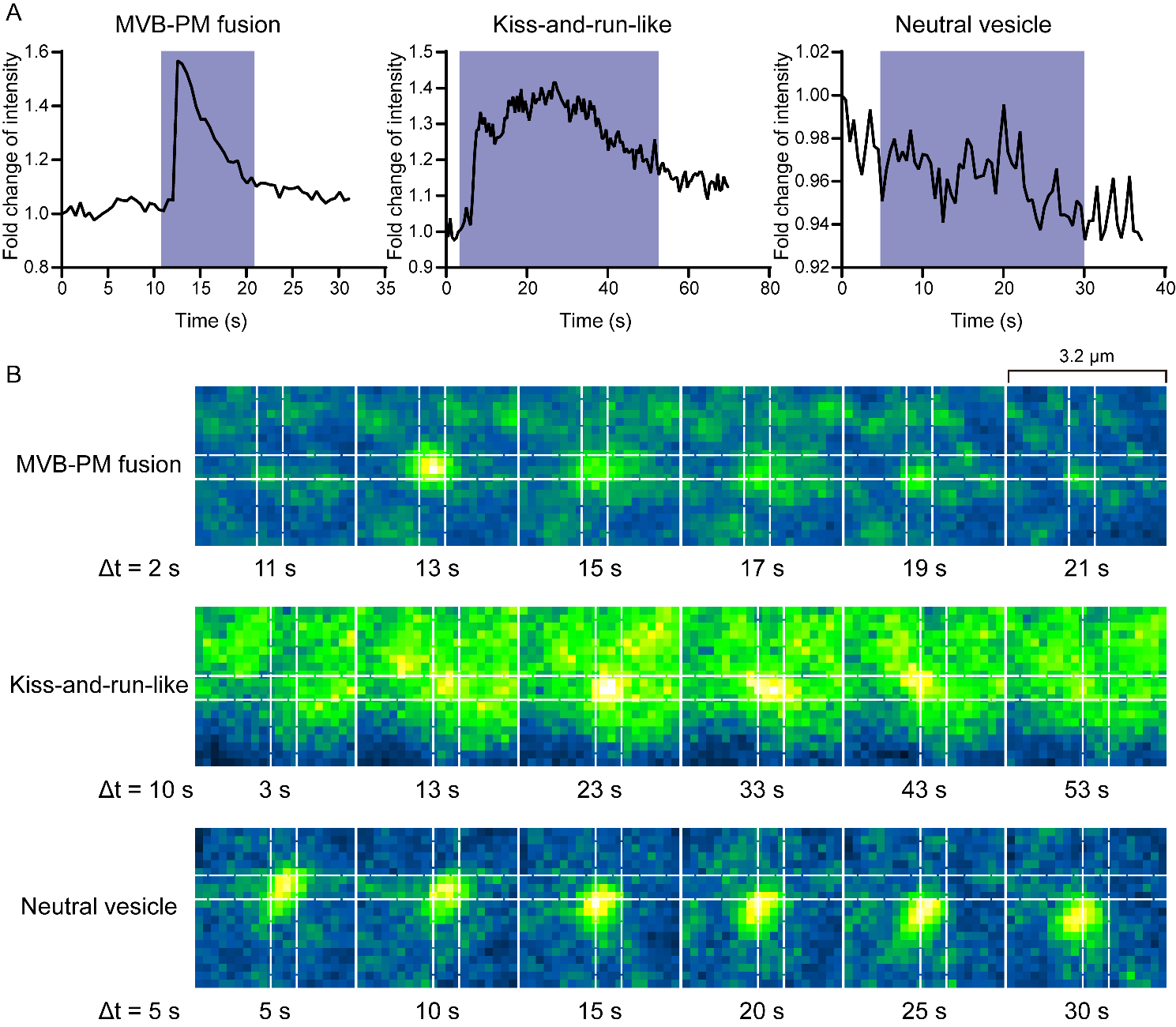


**Figure S1. Fluorescent features of different events characterized by TIRF microscopy. (A)** Representative fluorescence intensity profiles of an MVB–PM fusion event, a kiss-and-run-like event and an event of neutral vesicle trafficking, respectively. Purple block indicates the time course shown in (B). **(B)** Representative live-cell TIRF images of different events in diverse timescales.


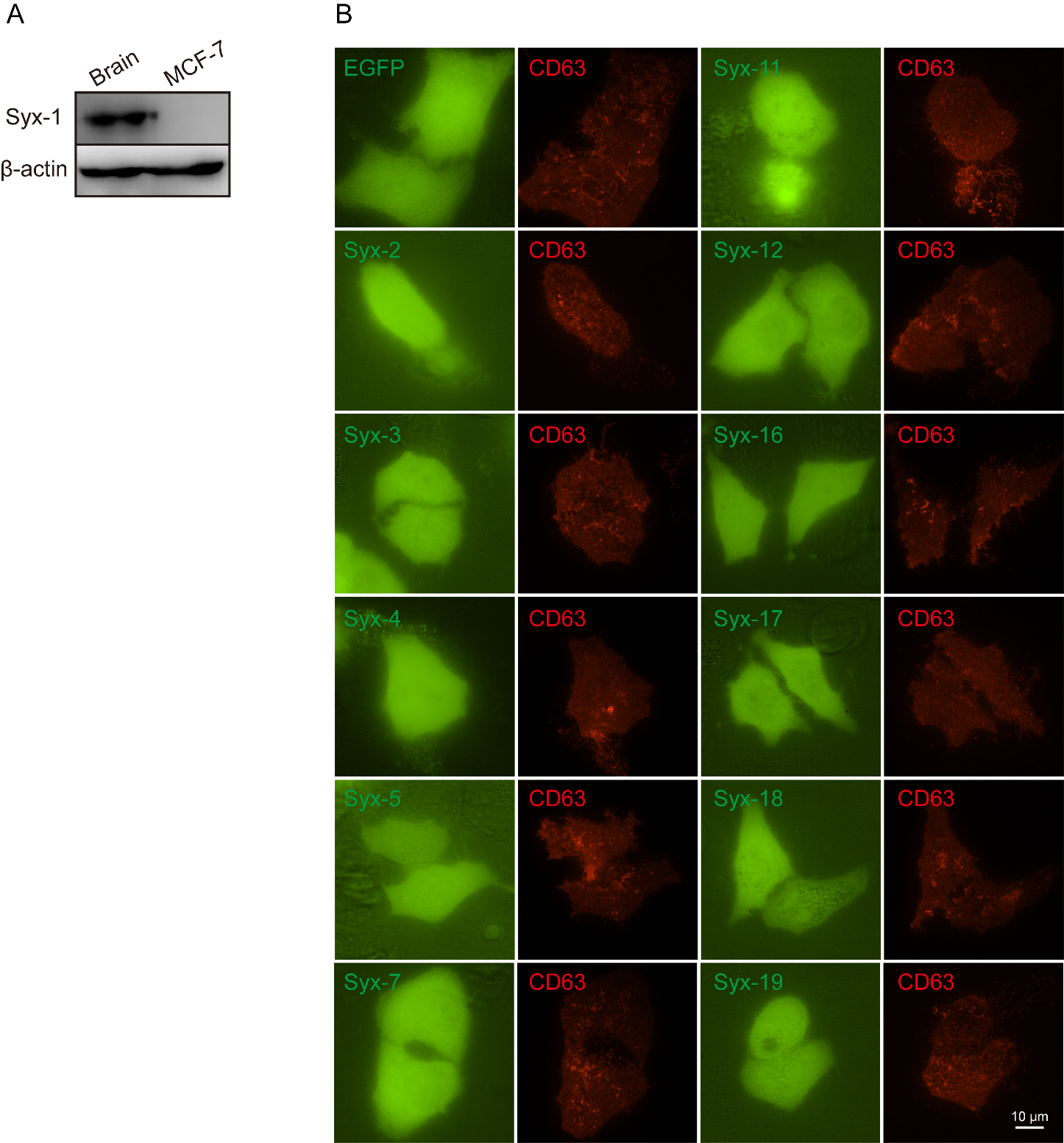


**Figure S2. Validation of co-expression of EGFP tagged cytoplasmic fragment of Q_a_-SNARE and CD63-mOrang in MCF-7 cells.** (A) Western analysis of Syx-1 in MCF-7 cells and in mouse brain homogenate, which serves as the positive control for Syx-1 detection. (B) EGFP fluorescence was excited by a halogen lamp in the wild-filed, and mOrange fluorescence was excited with a 532 nm laser in the TIRF filed. Scale bar, 10 μm.


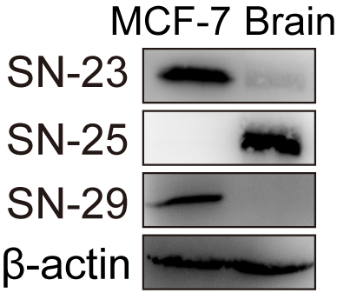


**Figure S3. SN-25 is not detectable in MCF-7 by western blot.** Western analysis of SN-23, -25 and -29 in MCF-7 cells and in mouse brain homogenate, which serves as the positive control for SN-25 detection.


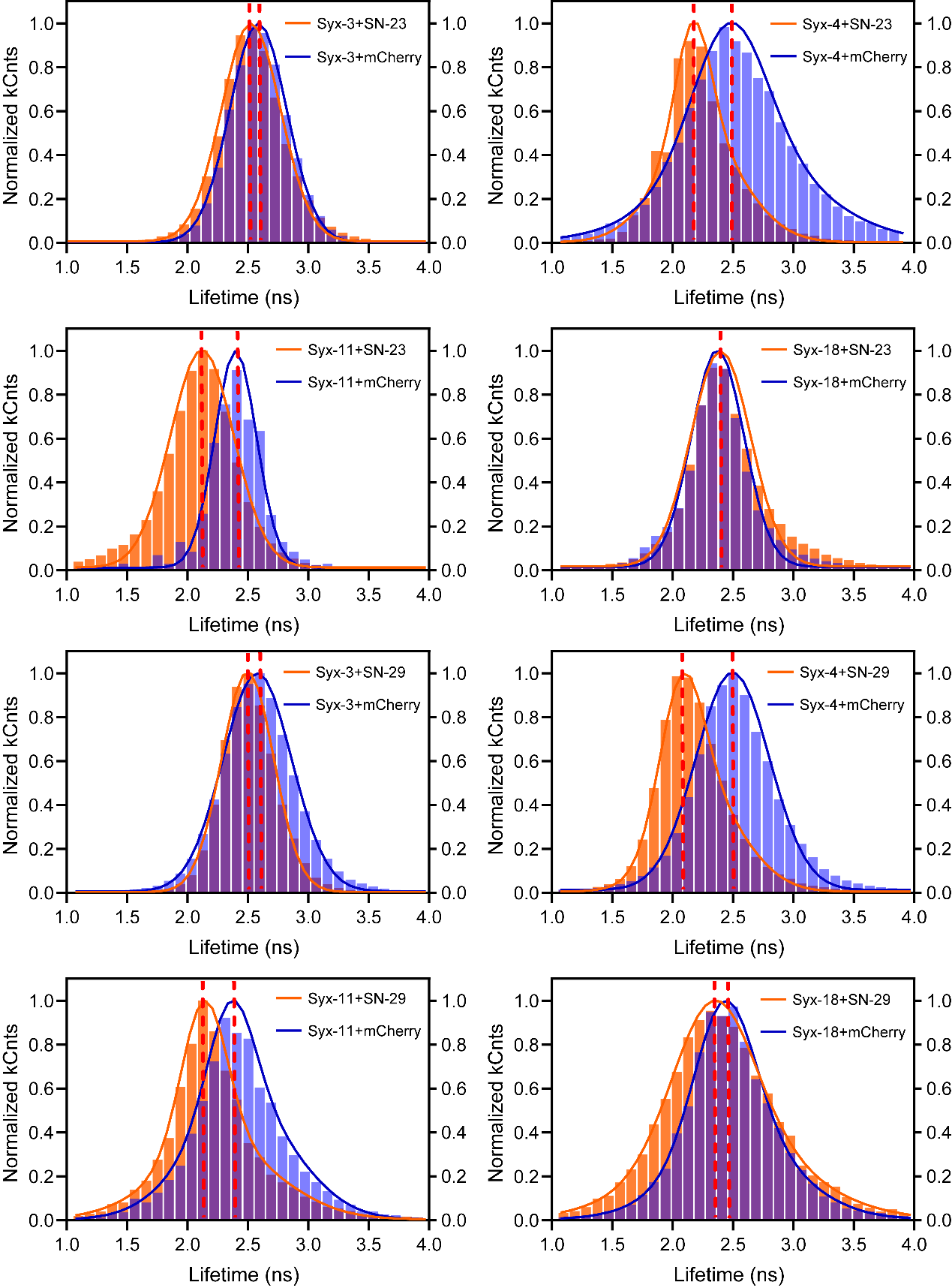


**Figure S4. Fluorescence lifetime distribution of EGFP-Syx-3, -4 -11 or -18 in the presence of mCherry and mCherry-SN-23 or -29.**

**
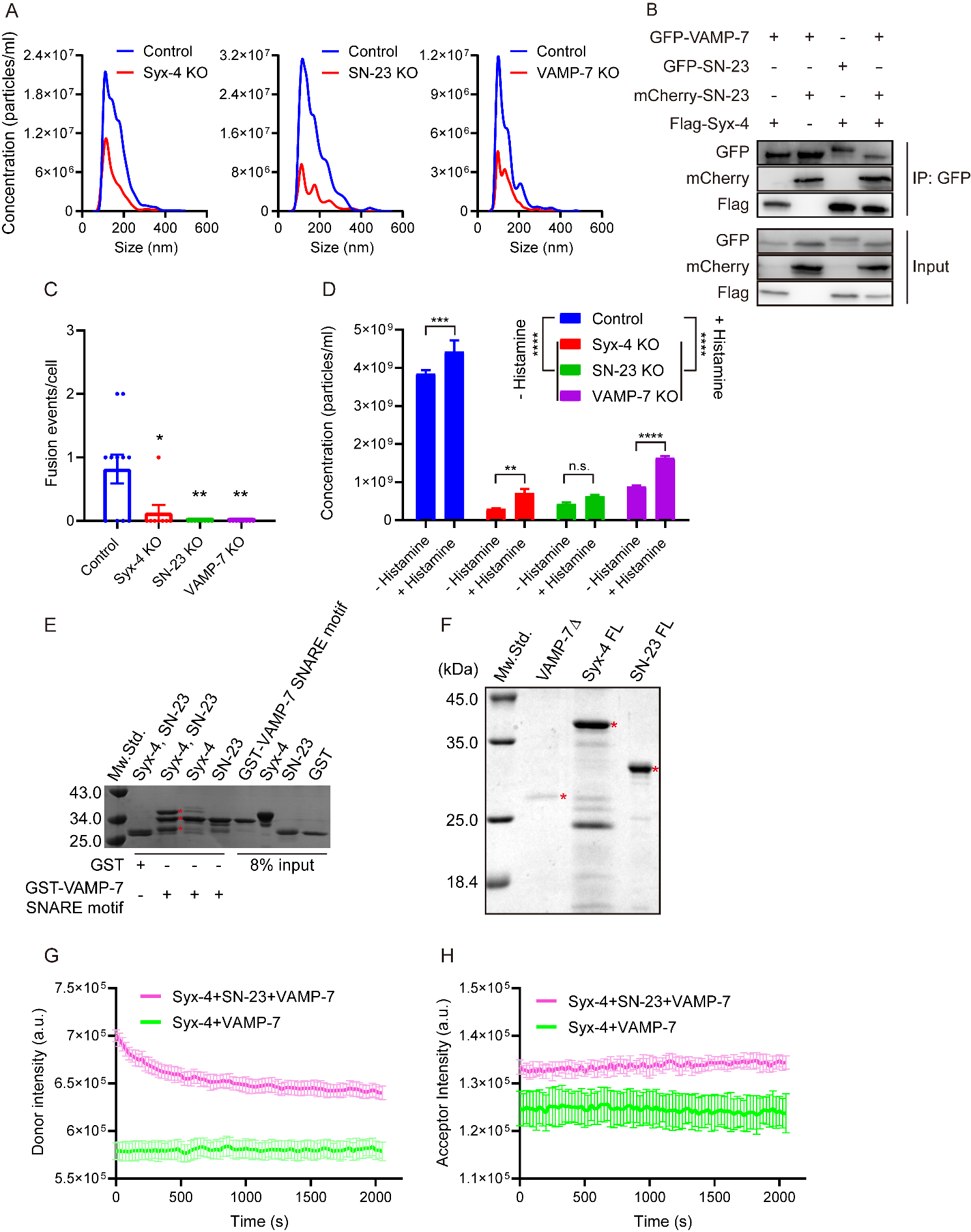
**

**Figure S5. Syx-4, SN-23 and VAMP-7 function in MVB–PM fusion.** **(A)** Representative NTA traces of sEVs from equal numbers of control and Syx-4, SN-23 or VAMP-7 knockout MCF-7 cells. **(B)** GFP-tagged proteins were immunoprecipitated by anti-GFP magnetic beads and the indicated coimmunoprecipitated SNAREs were analyzed by Western blot. **(C)** MVB–PM fusion activity of control, Syx-4, SN-23 or VAMP-7 knockout MCF-7 cells. 100 μm histamine was added prior to detection. Duration of TIRF imaging was 7 minutes for each cell. Data are presented as the means ± SEM, n ≥ 8 cells per condition, Mann Whitney test was used for data analysis, **p* < 0.05, ***p* < 0.01. **(D)** Quantification of sEVs isolated from the same volume of cell culture supernatants of equal numbers of control and Syx-4, SN-23 or VAMP-7 knockout MCF-7 cells with or without histamine treatment by NTA. Data are presented as the means ± SEM, n = 3 independent replicates, two-tailed t test, ***p* < 0.01, ****p* < 0.001, *****p* < 0.0001, n.s., no significance. **(E)** Coomassie stained gel from GST pull-down experiment. Red asterisks indicate that GST-VAMP-7 SNARE motif, Syx-4 (residues 1-274, the cytoplasmic fragment) and SN-23 assemble into a 1:1:1 complex. **(F)** Coomassie stained gel of purified proteins of VAMP-7 Δ12-3M, Syx-4 FL, SN-23 FL. FL, full-length. **(G and H)** Raw averaged fluorescence intensity traces of donor (G) and acceptor (H).

**
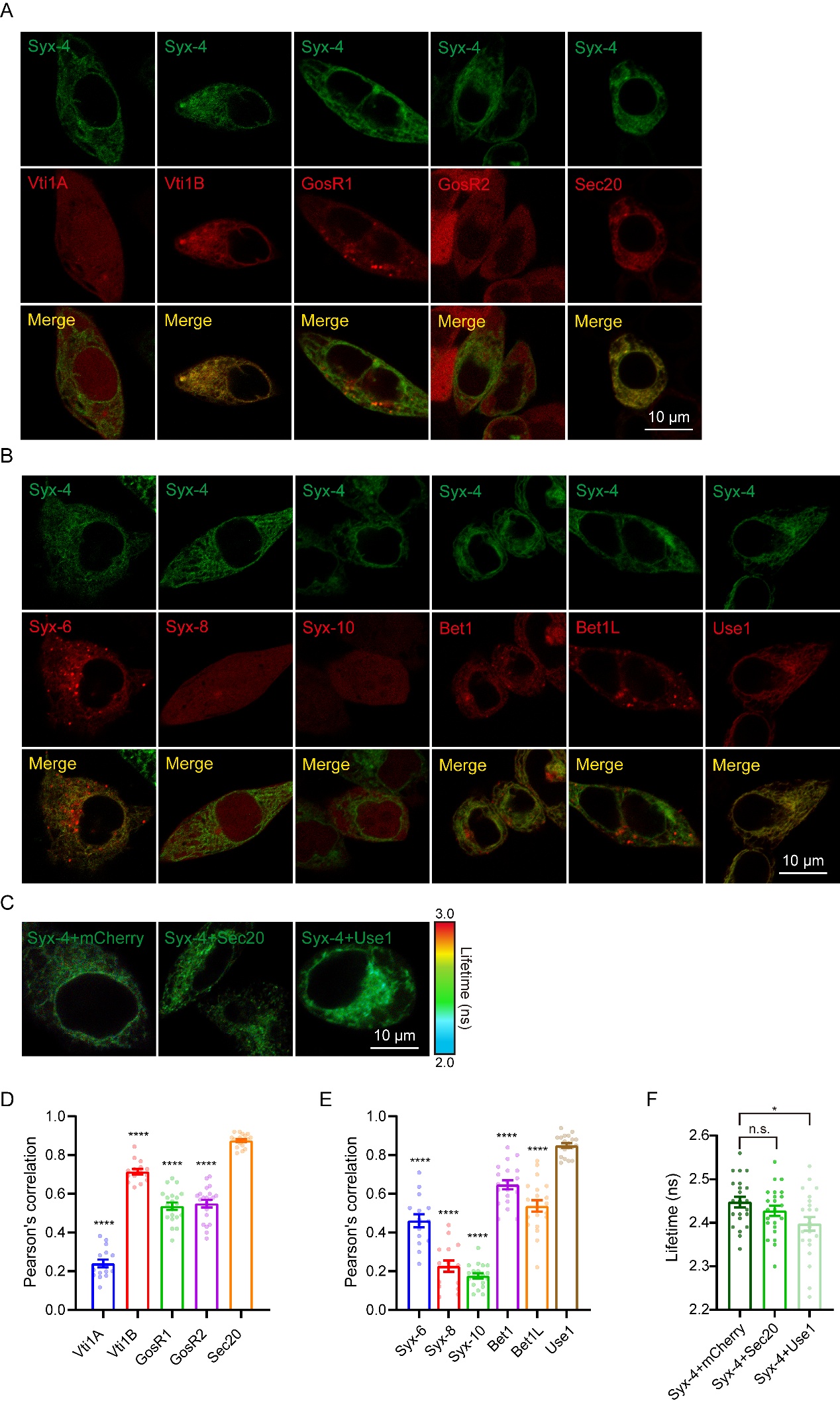
**

**Figure S6. Q_b_-SNARE Sec20 and Q_c_-SNARE Use1 colocalize with Syx-4, but the interactions between Sec20 or Use1 and Syx-4 are fairly limited.** **(A)** Representative confocal images of MCF-7 cells co-expressing EGFP-Syx-4 and mCherry-Vti1A, Vti1B, GosR1, GosR2 or Sec20, respectively. **(B)** Representative confocal images of MCF-7 cells co-expressing EGFP-Syx-4 and mCherry-Syx-6, -8, -10, Bet1, Bet1L or Use1, respectively. **(C)** FLIM images of MCF-7 cells co-expressing EGFP-Syx-4 and mCherry, mCherry-Sec20 or -Use1, respectively. Scale bar, 10 µm (A-C). **(D and E)** Pearson’s correlation between EGFP-Syx-4 and mCherry-Q_b_-SNAREs (D) or mCherry-Q_c_-SNAREs (E). **(F)** Averaged lifetime of EGFP-Syx-4 in the presence of mCherry, mCherry-Sec20 or -Use1, respectively. Data are presented as the means ± SEM, n ≥ 15 cells (D and E) or 22 cells (F) per condition from three separate replicates, ordinary one-way ANOVA, **p* < 0.05, *****p* < 0.0001, n.s., no significance (D–F).


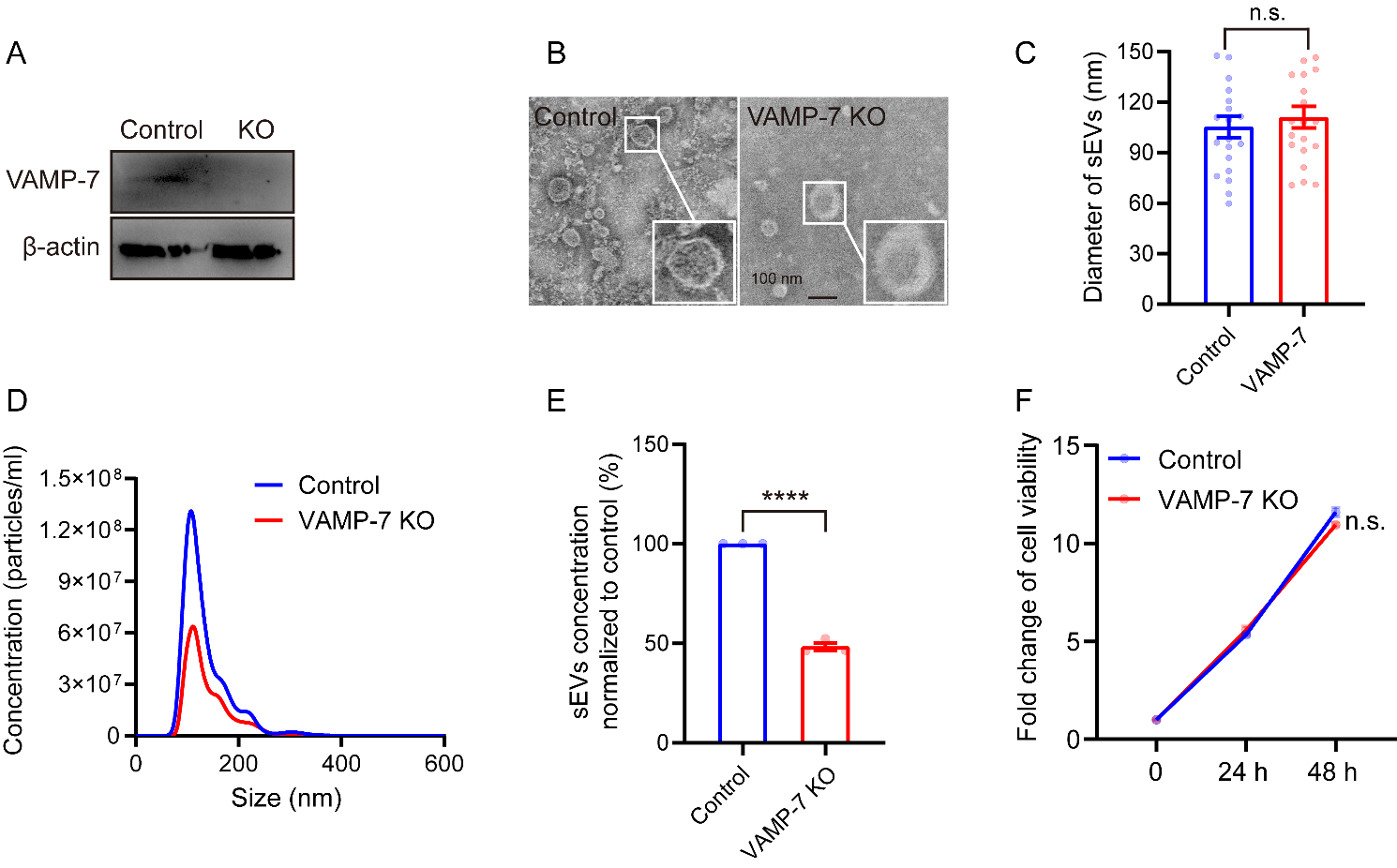


**Figure S7. VAMP-7 knockout suppresses exosome secretion in 4T1 cells. (A)** Validation of VAMP-7 knockout in 4T1 cells by western blot. **(B)** Representative TEM images of sEVs from control and VAMP-7 knockout 4T1 cells. **(C)** Diameter of sEVs from control and VAMP-7 knockout 4T1 cells by TEM. Data are presented as the means ± SEM, n = 20 sEVs, two-tailed t test, n.s., no significance. **(D)** Representative NTA traces of sEVs from equal numbers of control and VAMP-7 knockout 4T1 cells. **(E)** Quantification of sEVs isolated from the same volume of cell culture supernatants of equal numbers of control and VAMP-7 knockout 4T1 cells by NTA. Data are presented as the means ± SEM, n = 3 independent replicates, two-tailed t test, *****p* < 0.0001. **(F)** Cell viability of control and VAMP-7 knockout 4T1 cells detected by Cell Counting Kit-8. Data are presented as the means ± SEM, n = 6 independent replicates, two-tailed t test, n.s., no significance.

| Group | Pearson’s correlation |
| --- | --- |
| Syx-3+ SN-23 | 0.61±0.04 |
| Syx-4+ SN-23 | 0.75±0.03 |
| Syx-11+ SN-23 | 0.85±0.02 |
| Syx-18+ SN-23 | 0.08±0.01 |
| Syx-3+SN-25 | 0.44±0.03 |
| Syx-4+SN-25 | 0.60±0.02 |
| Syx-11+SN-25 | 0.71±0.01 |
| Syx-18+SN-25 | 0.08±0.01 |
| Syx-3+SN-29 | 0.47±0.03 |
| Syx-4+SN-29 | 0.72±0.02 |
| Syx-11+SN-29 | 0.83±0.03 |
| Syx-18+SN-29 | 0.32±0.02 |
| Syx-3+SN-47 | 0.17±0.01 |
| Syx-4+SN-47 | 0.32±0.02 |
| Syx-11+SN-47 | 0.35±0.03 |
| Syx-18+SN-47 | 0.32±0.02 |

**Table S1. Pearson’s correlation between Q_a_- and Q_bc_-SNARE.** Data are presented as the means ± SEM. Q_a_-SNARE refers to Syx-3, -4, -11 or -18, and Q_bc_-SNARE refer to SN-23, -25, -29 or -47.

| Group (Q_a+_Q_bc_) | Lifetime (ns) | | FRET efficiency (%) |
| --- | --- | --- | --- |
|  | EGFP-Q_a_+mCherry | EGFP-Qa+mCherry-Q_bc_ |  |
| Syx-3+SN-23 | 2.574±0.029 | 2.516±0.048 | 2.300±1.806 |
| Syx-3+SN-29 | 2.518±0.051 | 2.580±0.028 | 2.422±1.504 |
| Syx-4+SN-23 | 2.288±0.062 | 2.511±0.034 | 8.834±0.816 |
| Syx-4+SN-29 | 2.230±0.100 | 2.511±0.034 | 11.080±2.045 |
| Syx-11+SN-23 | 2.211±0.063 | 2.456±0.039 | 10.560±1.222 |
| Syx-11+SN-29 | 2.250±0.095 | 2.456±0.039 | 9.167±1.794 |
| Syx-18+SN-23 | 2.357±0.116 | 2.413±0.084 | 2.133±1.834 |
| Syx-18+SN-29 | 2.409±0.061 | 2.496±0.025 | 3.474±0.618 |

**Table S2. Lifetime of EGFP-Q_a_ SNAREs in the presence of mCherry or mCherry-Q_bc_-SNAREs, and FRET efficiency between EGFP-Q_a_ SNARE and mCherry-SN-23 or -29.** Data are presented as the means ± SEM. Q_a_-SNAREs refer to Syx-3, -4, -11 and -18, and Q_bc_-SNAREs refer to SN-23 and -29.

| Protein | Percentage of endogenous CD63-positive (%) | Percentage of exogenous CD63-positive (%) |
| --- | --- | --- |
| EGFP-VAMP-2 | 34.00±2.78 | 24.72±2.17 |
| EGFP-VAMP-3 | 15.21±1.89 | 11.88±2.32 |
| EGFP-VAMP-4 | 20.32±3.33 | 17.59±2.57 |
| EGFP-VAMP-7 | 82.72±1.71 | 65.07±1.96 |
| EGFP-VAMP-8 | 42.81±2.33 | 37.41±2.03 |
| EGFP-Ykt6 | 18.21±1.41 | 10.73±0.90 |
| CD63-mOrange | 92.39±0.76 | / |

**Table S3. Percentage of EGFP-R-SNARE-positive area that is CD63-positive.** Data are presented as the means ± SEM. R-SNARE refers to VAMP-2, -3, -4, -7, -8 and Ykt6.

| mCherry-Q_b_-SNARE | Pearson’s correlation | mCherry-Q_c_-SNARE | Pearson’s correlation |
| --- | --- | --- | --- |
| Vti1A | 0.24±0.02 | Syx-6 | 0.46±0.03 |
| Vti1B | 0.71±0.01 | Syx-8 | 0.23±0.03 |
| GosR1 | 0.54±0.02 | Syx-10 | 0.18±0.01 |
| GosR2 | 0.55±0.02 | Bet1 | 0.65±0.02 |
| Sec20 | 0.87±0.01 | Bet1L | 0.54±0.03 |
|  |  | Use1 | 0.85±0.01 |

**Table S4. Pearson’s correlation between EGFP-Syx-4 and mCherry-Q_b_-SNARE or -Q_c_-SNARE.** Data are presented as the means ± SEM. Q_b_-SNARE refers to Vti1A, Vti1B, GosR1, GodR2 or Sec20, and Q_c_-SNARE refers to Syx-6, -8, -10, Bet1, Bet1L or Use1.

| Group | Lifetime (ns) |
| --- | --- |
| Syx-4+mCherry | 2.448±0.01 |
| Syx-4+mCherry-Sse20 | 2.427±0.01 |
| Syx-4+mCherry-Use1 | 2.397±0.02 |

**Table S5. Lifetime of EGFP-Syx-4 in the presence of mCherry or mCherry-Sse20 or -Use1.** Data are presented as the means ± SEM.

**Video S1. Representative timelapse TIRF imaging of CD63-mOrange in the cell co-expressing CD63-mOrange and EGFP at 26 × normal speed with a 7-minute duration.**
